# *In situ* electrochemical regeneration of active 1,4-NADH for enzymatic lactic acid formation *via* concerted functions on Pt-modified TiO_2_/Ti†

**DOI:** 10.1039/d3sc04104b

**Published:** 2024-01-16

**Authors:** Nada H. A. Besisa, Ki-Seok Yoon, M. Yamauchi

**Affiliations:** a Department of Chemistry, Graduate School of Science, Kyushu University Fukuoka 819-0395 Japan yamauchi@ms.ifoc.kyushu-u.ac.jp; b International Institute for Carbon-Neutral Energy Research (WPI-I2CNER), Kyushu University Fukuoka 819-0395 Japan; c Institute for Materials Chemistry and Engineering (IMCE), Kyushu University Fukuoka 819-0395 Japan; d Advanced Institute for Materials Research (WPI-AIMR), Tohoku University Sendai 980-8577 Miyagi Japan; e Research Center for Negative Emissions Technologies (K-Nets), Kyushu University Fukuoka 819-0395 Japan

## Abstract

Nicotinamide adenine dinucleotide (NAD^+^) and its reduced form (NADH) are key cofactors serving as essential hydrogen acceptors and donors to facilitate energy and material conversions under mild conditions. We demonstrate direct electrochemical conversion to achieve highly efficient regeneration of enzymatically active 1,4-NADH using a Pt-modified TiO_2_ catalyst grown directly on a Ti mesh electrode (Pt-TOT). Spectral analyses revealed that defects formed by the inclusion of Pt species in the lattice of TiO_2_ play a critical role in the regeneration process. In particular, Pt-TOT containing approximately 3 atom% of Pt exhibited unprecedented efficiency in the electrochemical reduction of NAD^+^ at the lowest overpotential to date. This exceptional performance led to the production of active 1,4-NADH with a significantly high yield of 86 ± 3% at −0.6 V *vs.* Ag/AgCl (−0.06 V *vs.* RHE) and an even higher yield of 99.5 ± 0.4% at a slightly elevated negative potential of −0.8 V *vs.* Ag/AgCl (−0.2 V *vs.* RHE). Furthermore, the electrochemically generated NADH was directly applied in the enzymatic conversion of pyruvic acid to lactic acid using lactate dehydrogenase.

## Abstract 2

Nicotinamide adenine dinucleotide (NAD^+^) and its reduced form (NADH) are key cofactors serving as essential hydrogen acceptors and donors to facilitate energy and material conversions under mild conditions.

## Graphical abstract

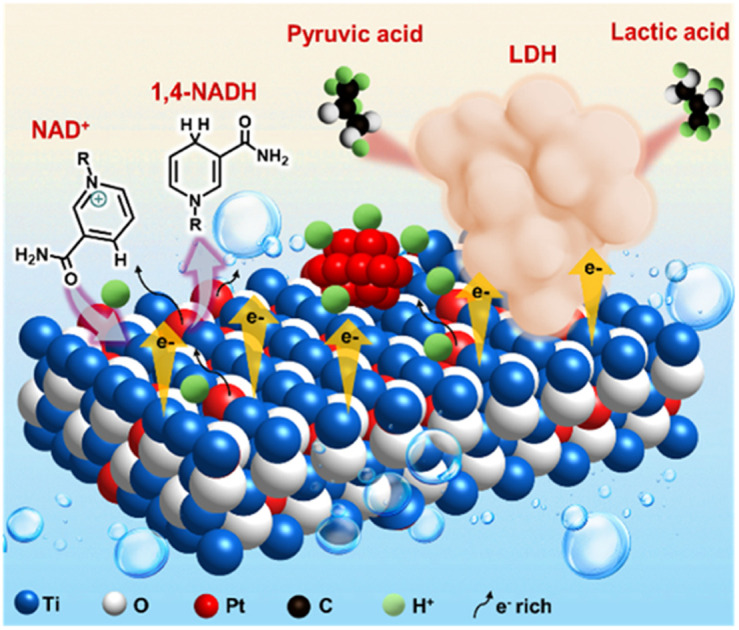

## Introduction

Enzymatic technology provides a highly efficient and environmentally friendly approach to the synthesis of a wide range of value-added chemicals, including chiral compounds, pharmaceuticals, and food additives, among other applications.^1–4^ Active enzymes are often accompanied by cofactors such as nicotinamide adenine dinucleotide (NAD), particularly its reduced form (NADH), which serves as a critical electron or hydrogen donor in both nature and industry.^3,5–7^

**Scheme 1:**
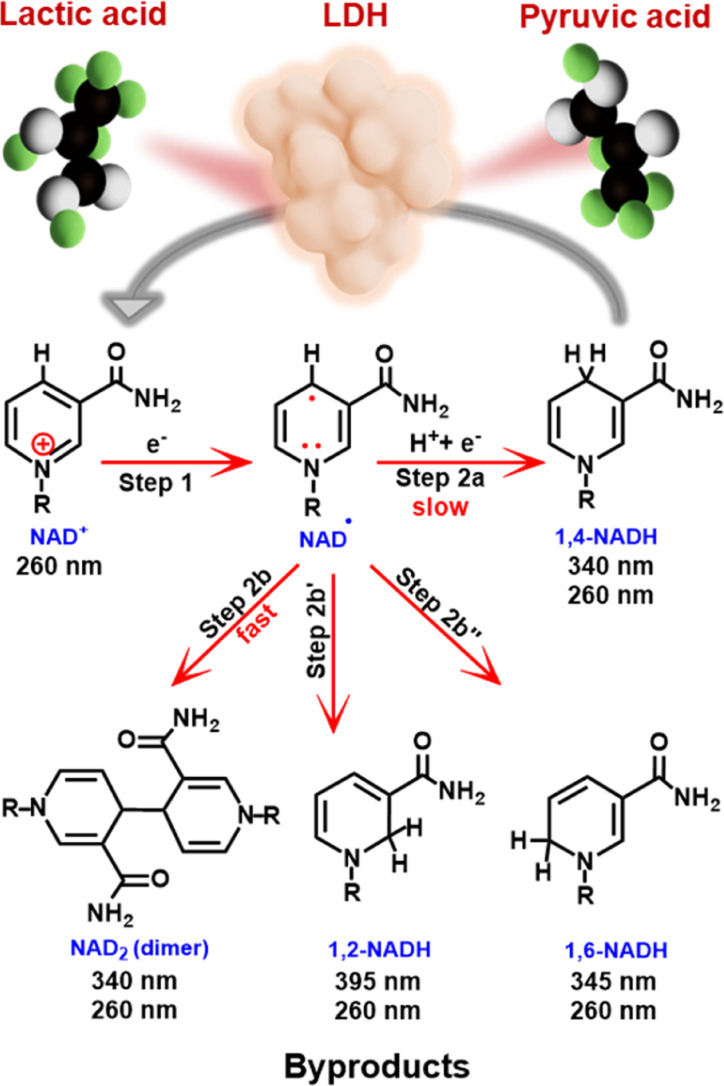
Mechanism of electrochemical reduction of NAD^+^, possible products and wavelengths of observable UV absorption peaks for each product. Application of the enzymatically active 1,4-NADH for lactic acid formation is also illustrated.

NADH is normally recycled through complex processes involving an electron transport chain such as glycolysis and the Krebs cycle, which has been a bottleneck preventing the widespread application of enzymatic systems. Therefore, more efficient cofactor recycling methods are highly desirable.^4^ Among various approaches, electrochemical hydrogenation of NAD^+^ to regenerate enzymatically active 1,4-NADH (NADH-reg, Scheme 1) has attracted considerable attention due to its simplicity and environmental friendliness.^7–10^ Indirect electrochemical systems often require mediators to facilitate electron transfer, resulting in lower overpotentials and avoidance of electrode fouling.^3^ However, these mediator systems suffer from the mediator instability and toxicity, affecting the final enzymatic product.^3,4^ In contrast, recent research on direct electrochemical hydrogenation systems for NADH-reg has shown an important advantage in eliminating the need for mediators.^3,5,11–16^

Both bare and modified electrodes have been employed for NADH-reg, but the yields remain unsatisfactorily low due to the preferential production of the enzymatically inactive forms (Scheme 1).^9^ NADH-reg proceeds in two steps:^8,17^ first, NAD^+^ is reduced to an NAD-free radical, and second, the free radical can either undergo protonation and further reduction to yield enzymatically active 1,4-NADH (Step 2a), or it can rapidly dimerize to form inactive products such as NAD_2_ (Step 2b) and other side products such as 1,2-NADH (Step 2b′) and 1,6-NADH (Step 2b′′). The kinetics of Step 2a for the formation of 1,4-NADH is significantly slower than that of the dimerization process.^8^ Therefore, efforts have been made to accelerate the second pathway, emphasizing the need for a smooth electrode-driven hydrogenation of the NAD free radical to achieve selective production of 1,4-NADH (Scheme S1a†). In this regard, the use of catalysts with high hydrogen evolution reaction (HER) capability has been a strategy to find a good electrocatalyst for NADH-reg.^8^ In addition, previous studies have reported high overpotentials and usage of unaffordable and high-cost materials,^8,10^ highlighting the demand for more practical electrode materials that can efficiently produce active 1,4-NADH at lower overpotentials.

**Scheme 2:**
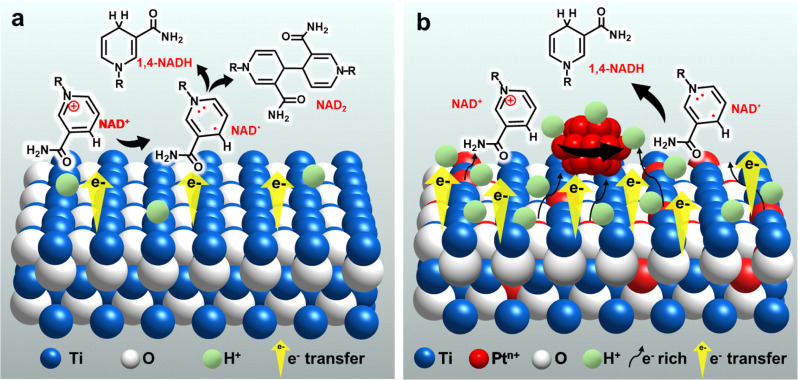
Suggested strategy and mechanism for the electrochemical reduction of NAD^+^ on (a) TOT and (b) Pt-TOTs.

Titanium dioxide (TiO_2_) has received much attention due to its abundance, non-toxicity, good stability, affordable cost.^18,19^ In addition, it has high activity in various applications, including electrochemistry.^20–24^ Recently, TiO_2_ has been found to exhibit high selectivity for the production of valuable products such as alcohols and amino acids from organic acids.^24–27^ Furthermore, sufficient controllability of its electronic states by doping with other elements is another advantage as an electrode material.^28–31^ Previous studies, including ours, have suggested that the unique performance of TiO_2_-based electrodes results from their ability to interact with carboxy groups of the reactants.^26,27,32,33^ Considering the polar nature of the amide moiety in NADs, analogous to the carboxy group, the TiO_2_ surface is expected to show feasible interactions with NADs and contribute to enhancing the selectivity for the production of NADH (Scheme 2a). Therefore, in this study, we chose TiO_2_ as an affordable and promising electrocatalyst to efficiently trap NAD^+^ and incorporation of Pt species, which shows excellent HER capability, and further promotes NAD^+^ hydrogenation to achieve highly selective and efficient electrochemical NADH-reg (Scheme 2b) for the advancement of sustainable and cost-effective enzymatic processes.

Here, we apply TiO_2_ grains grown on a Ti mesh cathode (TOT), and further introduce Pt into TOT by including a Pt source in a hydrothermal preparation process of TOT. The resultant Pt-modified TiO_2_/Ti cathode (Pt-TOT) was found to exhibit superior NADH-reg performance in terms of selectivity for 1,4-NADH, surpassing that of TOT and Pt electrodes. In-depth analyses of Pt-TOT revealed that the presence of Pt in various valence states on TiO_2_/Ti and defects plays a crucial role in enhancing the yield of 1,4-NADH (Scheme 2b) and lowering the overpotential for NADH-reg. Furthermore, the usability of the electrochemically generated 1,4-NADH in enzymatic lactic acid formation from pyruvic acid was then verified using lactate dehydrogenase (LDH), as shown in Scheme 1.

## Results and discussion

### Structural characterization of electrodes

Four Pt-modified TOT electrodes with different Pt contents were prepared using a hydrothermal process (see the ESI† for detailed methodology). The Pt concentration in the starting solution was adjusted to control the amount of Pt species in the electrodes. The resulting electrodes are denoted as Pt-TOT.*X* (*X* = 1–4), indicating increasing Pt content (Table S1†). To establish Pt contents in Pt-TOTs, inductively coupled plasma optical emission spectroscopy (ICP) was employed, and the obtained values are summarized in Table S1.†

**Fig. 1:**
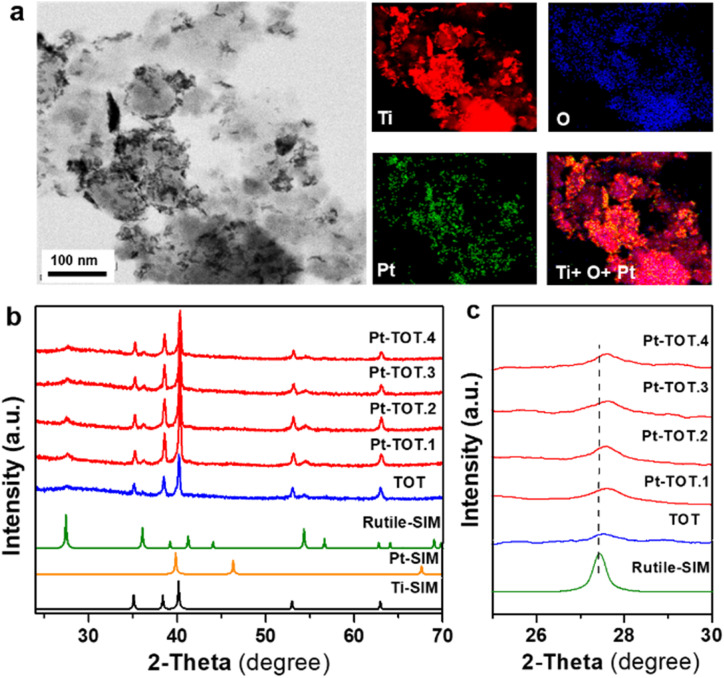
(a) STEM and EDS elemental mapping of Pt-TOT.2, (b) XRD pattern, and (c) expanded XRD pattern from 2*θ* = 24 to 30° of TOT and Pt-TOTs with different Pt content (the dashed line shows the diffraction peak position of rutile TiO_2_).

The morphology of prepared TOT and Pt-TOTs was investigated by transmission electron microscopy (TEM) and scanning electron microscopy (SEM) combined with energy dispersive X-ray spectroscopy (EDS, where Pt content was represented as Pt atom% according to EDS elemental analysis in this work) (Fig. 1a, S1–S3, and Table S2†). High contrast particles were observed on the surface of Pt-TOTs, the number of which increased proportionally with the Pt content (Fig. 1a and S1†). In TOT, undefined large grains measuring several tens of nanometers were observed (Fig. S2a†). In contrast, the grain size of Pt-TOTs decreased with increasing Pt content, as depicted in Fig. S2b–f.† Furthermore, EDS mapping analysis revealed the homogeneous distribution of Pt species throughout the grains of Pt-TOTs, with their density increasing with increasing Pt content (Fig. 1a and S3†). On the other hand, X-ray diffraction (XRD) patterns of TOT and Pt-TOTs primarily displayed weak and strong diffractions of tetragonal and hcp phases, respectively, indicating the formation of rutile-type (tetragonal) TiO_2_ grains on the hcp-type Ti mesh (Fig. 1b). Notably, no diffraction peaks corresponding to PtO or Pt metal phases were observed in Pt-TOTs. Rietveld analysis provided lattice constants for TiO_2_ grown on TOT and Pt-TOTs (Fig. S4 and Table S3†). Compared to TOT, the lattice constants a and b of Pt-TOTs slightly decreased with increasing Pt content, causing a subtle shift of diffraction peaks from the rutile phase toward the higher angle side (Fig. 1c). This observation suggests the substitution of 6-coordinated Ti^3+^ ions with a radius of 0.67 Å by Pt^4+^ ions with a smaller radius of 0.625 Å.^31^ Consequently, Pt species not only exist as nanoparticles on the surface but are also incorporated within the TiO_2_ lattice of Pt-TOTs.

### Electrochemical studies

**Fig. 2:**
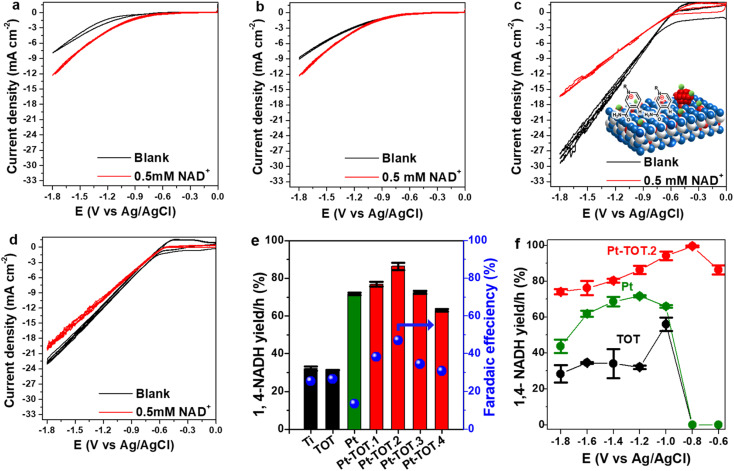
Cyclic voltammograms (CV) of (a) Ti, (b) TOT (c) Pt-TOT.2, the inset of which is a schematic representation of adsorption of NAD^+^, and (d) Pt in both blank (black) and 0.5 mM NAD^+^ solution (red), (e) yield and faradaic efficiency (FE) for NADH-reg at −1.2 V *vs.* Ag/AgCl, and (f) yield for NADH-reg on TOT, Pt, Pt-TOT.2 in a wide potential range.

To investigate onset potentials for the NAD^+^ reduction, we conducted cyclic voltammetry (CV) for the prepared electrodes over a potential range from 0 to −1.8 V *vs.* Ag/AgCl (−0.06 to −1.2 V *vs.* RHE) with a scan rate of 10 mV s^−1^ (Fig. 2a–d). In the absence of NAD^+^ (blank), the Ti mesh exhibited the most negative onset potential at approximately −1.1 V *vs.* Ag/AgCl (Fig. 2a). The onset potential on TOT was slightly shifted to approximately −1 V *vs.* Ag/AgCl (Fig. 2b), whereas Pt-TOT.2 and Pt showed even lesser negative onset potentials at −0.5 (Fig. 2c) and −0.6 V *vs.* Ag/AgCl (Fig. 2d), respectively, suggesting that the presence of TiO_2_ and Pt promotes both the HER and NAD^+^ reduction reaction. On the other hand, a larger negative current density (*j*) was observed in the presence of NAD^+^ on bare Ti mesh (Fig. 2a) and TOT (Fig. 2b) compared to the blank, suggesting the emergence of their catalytic activity for NAD^+^ reduction. Interestingly, Pt-TOT.2 showed the largest *j* in the absence of NAD^+^ (Fig. 2c), suggesting that inclusion of Pt with TiO_2_ enhances the HER. Notably, the *j* on Pt-TOT.2 in the presence of NAD^+^ was smaller than that in the blank, but larger than those on Ti and TOT. In addition, Pt exhibited a slightly larger *j* on the blank than in the presence of NAD^+^. Given that Pt is expected to show NADH-reg activity, the decrease in *j* on Pt-TOT.2 with NAD^+^ possibly implies the suppression of the HER due to NAD^+^ adsorption on the surface. Additionally, the current density in the presence of NAD^+^ followed the order of 20.2 mA cm^−2^ on Pt, 16.5 mA cm^−2^ on Pt-TOT.2, 12 mA cm^−2^ on TOT, and 10 mA cm^−2^ on Ti at −1.8 V *vs.* Ag/AgCl, possibly indicating that the coexistence of both Pt and TiO_2_ efficiently activates both the HER and NAD^+^ reduction.

We then conducted the electrochemical NAD^+^ reduction on the prepared electrodes using chronoamperometry (CA) at −1.2 V *vs.* Ag/AgCl (−0.6 V *vs.* RHE) where all electrodes showed the reduction current in the CV experiments with NAD^+^ using an H-type cell (Fig. S5†). As presented in Scheme 1, 1,4-NADH exhibits a UV absorption peak around 340 nm, whereas the peaks for possible side products such as (NAD)_2_ dimer, 1,6-NADH, and 1,2-NADH are observable at similar positions of 340, 345, and 395 nm, respectively. Therefore, we evaluated the yield for NADH-reg by enzyme activity assay (Scheme S1b†), in addition to calculating faradaic efficiency (FE) and the production rate for NADHs (C_NADH_ and C_1,4-NADH_, refer to the ESI for more details, Fig. S6†) to compare the selectivity on different electrodes. Fig. 2e shows that Pt and Pt-TOTs exhibit larger yields for NADH-reg than Ti and TOT, suggesting that the Pt loading significantly enhances NADH-reg. Interestingly, Pt-TOT.1, Pt-TOT.2 and Pt-TOT.3 demonstrated higher NADH-reg yields than Pt, implying that the presence of TOT is crucial for achieving higher selectivity for NADH-reg. As a result, Pt-TOT.2 showed the largest yield for NADH-reg of 86 ± 3% with the highest FE of 47% (Fig. 2e). Further increase in Pt content led to a slight decrease in catalytic activity. We then presume that NADH-reg preferentially occurs around Pt sites interacting with TiO_2_, whereas the HER surpasses NADH-reg once the number of Pt nanoparticles exceeds a level to form HER sites isolated from influence from the TiO_2_ surface.

Fig. 2f compares NADH-reg yields on Pt, TOT and Pt-TOT.2 over a wide range of potentials. Below −1.2 V *vs.* Ag/AgCl, the yields for NADH-reg on TOT and Ti electrodes were approximately 30% (Fig. 2f and S7a†). In contrast, at a less negative potential of −1.0 V *vs.* Ag/AgCl, the NADH-reg performance on TOT and Ti was enhanced, resulting in yields of 56 ± 4 and 68 ± 1%, respectively. However, at potentials less negative than −1 V *vs.* Ag/AgCl, no NADH-reg was observed on either Ti or TOT. Similarly, NADH-reg on Pt started at −1 V and showed the maximum with yield of 72 ± 1.5% at −1.2 V *vs.* Ag/AgCl. In contrast, Pt-TOT.2 demonstrated a significantly high yield of 86 ± 3% at only −0.6 V *vs.* Ag/AgCl (−0.06 V *vs.* RHE), which is the lowest overpotential among all those reported so far (Table S4†). At a slightly more negative potential of −0.8 V *vs.* Ag/AgCl (−0.2 V *vs.* RHE), the highest yield of 99.5 ± 0.4% for NADH-reg was achieved on Pt-TOT.2, combined with high stability and reusability over the number of cycles (Fig. S7b†). In addition, it showed the highest current density at −0.8 V *vs.* Ag/AgCl confirming the high activity towards NAD^+^ reduction (Fig. S7c†).

**Fig. 3:**
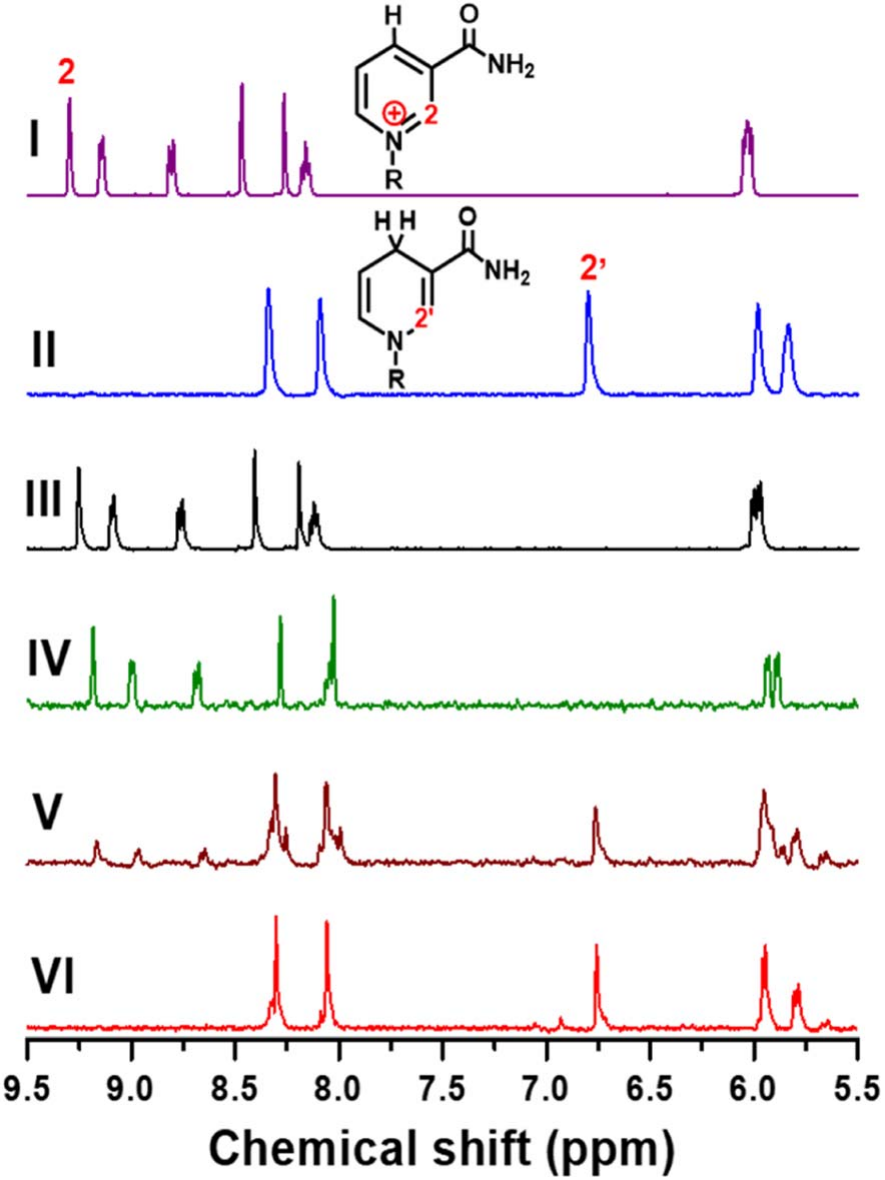
^1^H NMR spectra of NAD^+^ (4 mM in 0.1 M D_2_O phosphate buffer, pH 6) (I), 1,4-NADH (4 mM in 0.1 M D_2_O phosphate buffer, pH 6) (II), products generated from 4 mM NAD^+^ in 0.1 M D_2_O phosphate buffer with pH 5.8 at −0.8 V *vs.* Ag/AgCl on TOT (III) for 2 h, Pt (IV) for 2 h, and Pt-TOT.2 (V) for 2 h, and on Pt-TOT.2 (VI) for 4 h.

To further confirm the product selectivity, ^1^H NMR measurements were performed for products generated in CA at −0.8 V *vs.* Ag/AgCl with 4 mM NAD^+^ solution, which is 8 times higher than the concentration used in previous CA measurements to obtain sufficient NMR intensity. ^1^H NMR spectra of pure commercial NAD^+^ and 1,4-NADH as well as the products of our electrochemical NADH-reg on Pt, TOT, and Pt-TOT.2 are shown in Fig. 3. A singlet signal located at 9.28 ppm is characteristic of the H-2 hydrogen on the NAD^+^ structure of the nicotinamide moiety (Fig. 3I). The two singlets observed between 8 and 8.5 ppm are related to the hydrogen atoms on the adenine ring contained in the R group. In contrast, freshly prepared 1,4-NADH solution showed a singlet located at 6.8 ppm which is characteristic for the H-2′ of the nicotinamide ring of NADH and two singlets related to the adenine ring (Fig. 3II). ^1^H NMR spectra of the products on Pt and TOT presented signals assignable to NAD^+^ only, confirming the inability of Pt and TOT to produce active NADH at −0.8 V *vs.* Ag/AgCl (Fig. 3III and IV). On the other hand, the characteristic singlet at 6.8 ppm, assignable to the production of 1,4-NADH, was observed at more negative potential than −1.2 V *vs.* Ag/AgCl on Pt although accompanying a singlet at 7 ppm suggesting the formation of 1,6-NADH^34^ (Fig. S7d†). Surprisingly, Fig. 3V shows an obvious singlet signal at 6.8 ppm, confirming the 1,4-NADH formation in addition to signals of the starting NAD^+^. The results suggest that only pure 1,4-NADH is produced on Pt-TOT.2 without formation of other side products. To completely convert the starting NAD^+^, longer-time reaction (4 h) was conducted. Although signals for starting NAD^+^ almost disappeared and signals for 1,4-NADH grew, a small singlet was detected at 6.93 ppm (Fig. 3VI) with the integral ratio to that for 1,4-NADH of 0.086 : 1. According to the previous report,^34^ the 6.93 ppm peak is assignable to the formation of 1,6-NADH. This phenomenon can be understood by considering considerably high concentration of starting NAD^+^ compared to that in CA, which led to higher coverage of intermediates and induces formation of the side products.^35^ Thus, application of appropriate reaction conditions is critical to improve selectivity to produce 1,4-NADH.

### Mechanistic understanding

**Fig. 4:**
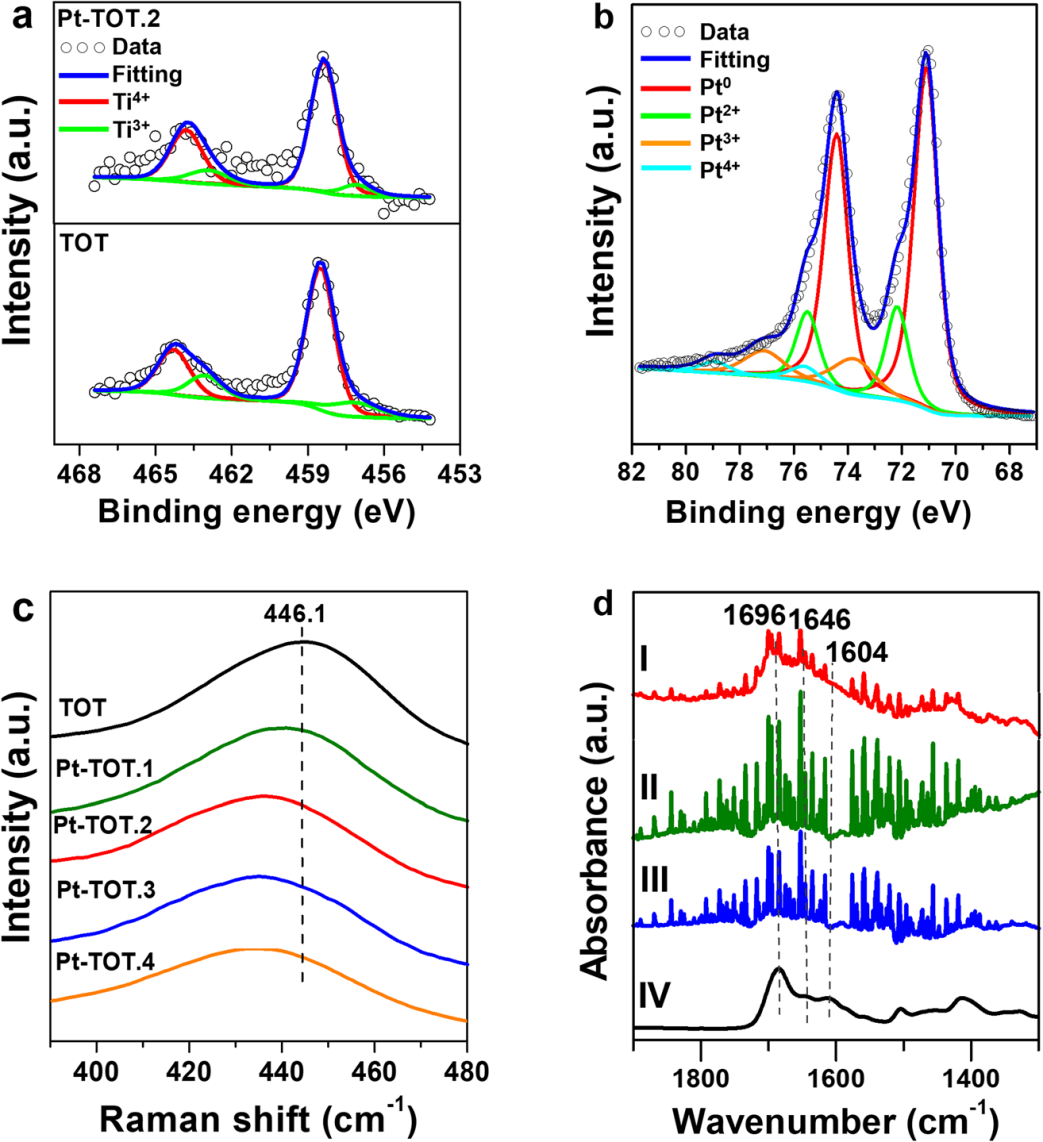
(a) Ti 2p XPS spectra of TOT and Pt-TOT.2, (b) Pt 4f XPS spectra of Pt-TOT.2, (c) Raman spectra and of TOT and Pt-TOTs in the range from 390 to 480 cm^−1^, and (d) FT-IR of adsorbed NAD^+^ on the surface of (I) Pt-TOT.2, (II) TOT (III) Ti, and (IV) ATR-IR spectrum of the NAD^+^ crystal (95%).

To gain insight into the catalytic performance of our electrodes, we conducted XPS measurements to investigate the electronic properties of the prepared TOT and Pt-TOTs (Fig. 4, S8, and Table S5†). Interestingly, Fig. 4a and S8† revealed that increasing Pt content in Pt-TOTs resulted in a slight positive shift of the peaks of both Ti 2p and O 1 s signals compared to those on TOT.^31^ This shift probably indicates the interaction of Pt species with Ti and O, forming Ti–O–Pt bonds and suggesting an increase in defects (Ti^3+^ and oxygen vacancies, V_o_) as presented in Table S5.† The XPS Pt 4f spectra of Pt-TOT.2 suggested the presence of Pt^*n*+^ species with different valence states in Pt-TOT.2 (Fig. 4b, ESI†).^31^ Notably, the incorporation of Pt resulted in a red shift of the Raman peak from 446.1 cm^−1^ to a lower wavenumber (Fig. 4c and S9†). This red shift can be attributed to the introduction of defects and an increase in V_o_, likely due to the replacement of Ti by Pt. The Raman and XPS analyses collectively suggest that Pt species occupy certain Ti^3+^ positions and promote Ti–O–Pt interactions.^31^

To further examine the role of TOT in NAD^+^ adsorption, we conducted Fourier transform infrared spectroscopy using an attenuated total reflectance configuration (ATR-IR) for analysis of adsorbed NAD^+^ on Ti, TOT, and Pt-TOT.2 surfaces after immersing into 0.5 mM NAD^+^ solution, washing, and drying (Fig. 4d). We observed broad signals on all electrodes at wavenumbers similar to the reported values, with sharp signals originating from the electrolytes (water vapor). We also measured the IR spectrum of the NAD^+^ crystal (95%) and observed broad peaks around 1690, 1646, and 1608 cm^−1^, corresponding to signals observed at 1696 (C=O stretch, nicotinamide), 1646 (NH_2_ bend, adenine), and 1604 cm^−1^ (C5–C6 stretch, adenine) for NAD^+^ powder.^15*b*^ By comparing these results, all spectra seem to contain spectral components of NAD^+^ and Pt-TOT.2 has the largest signals assignable to NAD^+^, possibly indicating that favorable environments for NAD^+^ adsorption are created on Pt-TOT.2, probably due to cooperative interactions between TOT and Pt. In particular, this may be attributed to the presence of oxygen vacancies formed through Ti–O–Pt interactions, which facilitate the adsorption of the amide C=O.^15*c*^ The existence of various valence states on Pt species has been previously correlated with the favorable electron transfer,^31^ and the formation of linking Ti–O–Pt bonding networks in the oxide and the boundary around Pt nanoparticles on the surface can mediate the electron transfer to the adsorbed NAD^+^ (Scheme 2b).^36^ These synergistic effects contribute to the remarkable catalytic performance of Pt-TOTs in NADH-reg and provide valuable insights for the design of efficient electrochemical processes.

We further investigated the electrochemical double layer capacitance (*C*_dl_) on Pt and Pt-TOT.2 to gain deeper insights into the electrode surfaces. The *C*_dl_ was evaluated based on the slope of the charging current, as shown in Fig. S10.† Subsequently, the corresponding electrochemical surface areas (ECSAs) were calculated from the *C*_dls_ and are presented in Fig. S11d.† Among the Pt-containing electrodes, it is evident that Pt-TOT.2 exhibits the highest values for both electrochemical double layer capacitance (*C*_dl_) and electrochemical surface area (ECSA). The performance of the hydrogen evolution reaction (HER) on these electrodes at −0.8 V *vs.* Ag/AgCl was also assessed as shown in Fig. S10d.† Remarkably, Pt-TOT.2 demonstrated the highest partial current density for the HER among the three electrodes, whereas Pt exhibited the lowest HER. We think that the formation of different valence states of Pt (Pt^*n*+^, *n* = 0, 2, 3, or 4) plays an important role in enhancing the NADH-reg.

This observed trend provides a straightforward explanation for the behavior in NADH-reg; Pt-TOTs likely possess the largest number of hydrogens adsorbed on their surface (H_ads_), a favorable condition for enhancing the kinetics of Step 2a in Scheme 1 (for more detailed explanation of the HER, refer to the ESI†). Furthermore, Pt-TOTs predominantly adsorb NAD^+^, making them an ideal platform for both NAD adsorption and efficient hydrogen supply. The interplay of TiO_2_ in NAD^+^ adsorption and the presence of Pt^*n*+^ species in Pt-TOTs, responsible for enhancing H_ads_ coverage and promoting electron transfer to the adsorbed NAD molecules, collectively contribute to accelerating the 1,4-NADH production. Notably, the rapid hydrogenation of NAD molecules on Pt-TOTs effectively limits the occurrence of dimerization processes and side products, ensuring superior selectivity.

In light of these results, we have presented a plausible mechanism for NADH-reg on TOT and Pt-TOTs, which is illustrated in Scheme 2. The findings presented in this study shed light on the mechanism underlying NADH-reg on Pt-TOTs, and we propose the mechanism based on our results in the ESI† (eqn (S14)–(S19). Therefore, Pt-TOTs emerge as a highly promising electrode for efficient electrochemical regeneration of enzymatically active 1,4-NADH at significantly low overpotentials.

**Fig. 5:**
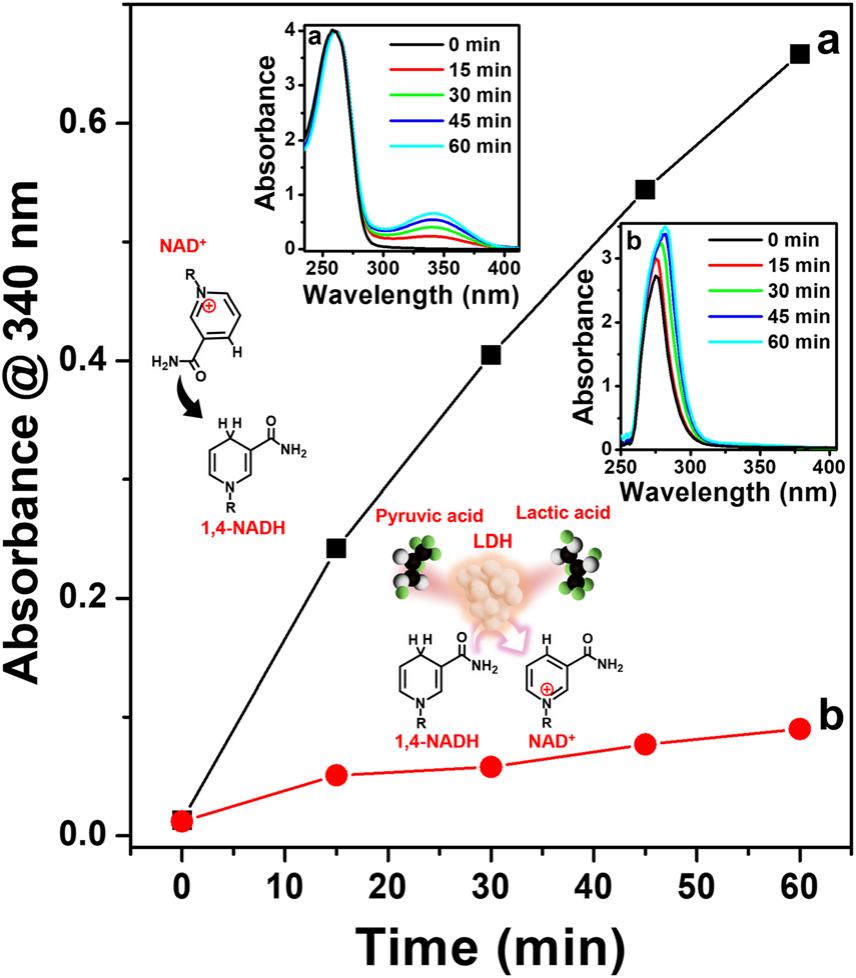
UV absorbance at 340 nm plotted against time for the electrochemical reduction of (a) 0.25 mM NAD^+^, and (b) 0.25 mM NAD^+^ with 20 mM pyruvic acid and 8 U ml^−1^ LDH from 0 min to 60 min. Insets show the corresponding UV absorbance measured for samples taken every 15 min during the electrochemical reduction of (a) and (b) and schematic representation of the reactions involved.

Furthermore, we evaluated the enzymatic activity of the 1,4-NADH produced using Pt-TOT.2 for the electrochemical generation of lactic acid from pyruvic acid. For this purpose, we utilized purified lactate dehydrogenase (LDH) from *Lactobacillus acidophilus* as the enzyme in our system (refer to ESI, Fig. S11 and S12†), along with the addition of pyruvic acid to the NAD^+^ solution. To monitor the production of lactic acid, we employed UV spectrophotometry and an H-type electrochemical cell, analyzing the product at 15-minute intervals (Fig. 5). In addition, we employed *in situ* UV spectroscopy (Fig. S13†) to confirm the consumption of NADH produced by our system during the lactic acid formation over time (Fig. S14†). Our observations revealed a progressive increase in the concentration of generated 1,4-NADH over time during the electrochemical reaction in the absence of pyruvic acid and LDH, as shown in Fig. 5, the insets (a) and (b), and S14.† Conversely, when we introduced both pyruvic acid and LDH, the concentration of 1,4-NADH became negligible, indicating that the produced 1,4-NADH was immediately utilized by LDH for the reduction of pyruvic acid (Fig. 5 and S14†). Furthermore, we performed HPLC analysis (Fig. S15†) to verify the product generated from the enzymatic electrochemical formation of lactic acid on Pt-TOT.2, which conclusively confirmed the production of lactic acid. These results unequivocally demonstrate that the generated 1,4-NADH using the Pt-TOT.2 electrode effectively serves as an electron donor in the enzymatic reaction catalyzed by LDH.

## Conclusion

Pt-TOTs demonstrated exceptional performance in NADH-reg. Notably, Pt-TOT.2 exhibited the highest yield of 99.5 ± 0.4% at the lowest reported overpotential of only −0.8 V *vs.* Ag/AgCl (−0.2 V *vs.* RHE), and 86 ± 3% at an even lower overpotential of −0.6 V *vs.* Ag/AgCl (−0.06 V *vs.* RHE). This outstanding performance arises from the synergistic effects of the predominant NAD adsorption on TiO_2_ and the rapid kinetics of hydrogenation achieved on Pt-TOT.2, with the presence of Pt^*n*+^ species in different valence states. The integration of such synergistic surface functions at the electrode interface emerges as a pivotal factor in achieving efficient electrochemical processes. Moreover, we validated the enzymatic activity of the 1,4-NADH produced using our designed electrode by converting pyruvic acid to lactic acid using LDH. The highly efficient NADH-reg demonstrated in this study holds great promise for advancing enzymatic systems and facilitating their widespread application in energy and materials transformations.

## Data availability

We provided the related data in the ESI.†

## Author contributions

Nada H. A. Besisa: conceptualization, investigation, experimental, writing – original draft, validation, analytical interpretation. Ki-SeokYoon: review and editing. M. Yamauchi: conceptualization, review and editing, supervision.

## Conflicts of interest

There are no conflicts to declare.

## Supplementary Material

SC-015-D3SC04104B-s001
