# Supplementary material for: In situ electrochemical regeneration of active 1,4-NADH for enzymatic lactic acid formation via concerted functions on Pt-modified TiO2/Ti
Source: Chem Sci. 2024 Jan 16;15(9):3240–8. doi: 10.1039/d3sc04104b (PMC10901512; doi:10.1039/d3sc04104b)
Supplement: SC-015-D3SC04104B-s001 [file SC-015-D3SC04104B-s001.pdf]

Electronic Supplementary Material (ESI) for Chemical Science.
This journal is © The Royal Society of Chemistry 2024

Supporting Information

In situ electrochemical regeneration of active 1,4-NADH for enzymatic lactic acid formation via concerted functions on Pt-modified TiO_2/Ti

Nada H A Besisa[a], Ki-SeokYoon[b], M. Yamauchi[a,b,c,d,e]*

[a]Department of Chemistry, Graduate School of Science, Kyushu University, Fukuoka 819-0395, Fukuoka, Japan. [b]International Institute for Carbon-Neutral Energy Research (WPI-I^{2}CNER), Kyushu University, Fukuoka 819-0395, Fukuoka, Japan. [c]Institute for Materials Chemistry and Engineering (IMCE), Kyushu University, Fukuoka 819-0395, Fukuoka, Japan. [d]Research Center for Negative Emissions Technologies (K-Nets), Kyushu University, Fukuoka 819-0395, Fukuoka, Japan. [e]Advanced Institute for Materials Research (WPI-AIMR), Tohoku University, Sendai 980-8577, Miyagi, Japan.

EXPERIMENTAL

Materials. Ti mesh (100 mesh) was purchased from Manabe industry co. Pt coil and Pt sheet were purchased from BAS Inc. Toray carbon paper and GDL paper were purchased from Alfa Aesar. Co. Nafion NRE-212 was purchased from Sigma-Aldrich Co. Nicotinamide-adenine dinucleotide reduced form was purchased from Tokyo Chemical Industry Co., LTD, while, nicotinamide-adenine dinucleotide oxidized form, and alcohol dehydrogenase were purchased from Oriental Yeast Co., LTD. Hydrogen hexachloro platinate (IV) hexahydrate, potassium phosphate, N,N-dimethylformamide, lithium acetate dihydrate, acetic acid, acetaldehyde, were purchased from Fujifilm Wako Pure Chemical Japan Co., LTD. Lactobacillus acidophilus bacteria was purchased from National Institute of Technology and Evaluation (NITE).

Preparation of electrodes. Pt-doped TiO_2 grown on Ti electrodes (Pt-TiO_2/Ti) with different Pt contents have been prepared through the in situ hydrothermal process inspired from the synthesis route reported in Ref. 1, with some modifications. In the current study, Ti mesh was used as a substrate. Ti mesh was cut into 2×2 cm^2 pieces (geometric area= 8 cm^2), then ultrasonically cleaned in a mixture of deionized water, ethanol, and acetone with equivalent ratios. The cleaned Ti pieces were then added into a homogeneous and clear mixture of dimethylformamide (DMF) as a solvent, Lithium acetate dihydrate (LiAc.2H_2O), acetic acid (HAc) as a buffer solution, and different amounts of hydrogen hexachloroplatinate (IV) hexahydrate (H_2PtCl_6, 1.0gm/ 100ml) and followed by autoclaving at 200℃ for 24 h. Afterwards, the produced pieces were washed with water and ethanol several times, and then completely dried. The prepared electrodes were named **Pt-TOT.X**, where X is given numbers from 1 to 4 according to the loading amount of Pt (weight%) to whole catalyst as shown in Table S1. TiO_2 grown on Ti was prepared with the same manner without H_2PtCl_6 aqueous solution and is indicated as TOT.

Furthermore, a pure Pt coil (Pt, geometric area= 3.6 cm^2) and Pt sheet (geometric area= 8 cm^2) were also used as working electrodes for comparison.

Measurement and characterization. X-ray diffraction (XRD) patterns of prepared electrodes were measured using Rd2-phaser (Bruker) with Cu-Kα radiation (λ= 1.54184Å). Field emission scanning electron microscopy (FESEM) images were taken with JSM7900F (JEOL). Transmission electronic microscopy (TEM) on JEMARM200CF (JEOL) operated at 200 kV was employed to show the morphologies. Raman spectra were measured using a confocal remote Raman spectrometer (MB11UF000000097.000) equipped with a 532 nm LD/YAG laser. X-ray photoelectron spectroscopy (XPS) analysis was conducted on PHI5000 versa probe (UlVAC-PHI) using Al-Kα radiation (1486.6ev). The contaminant carbon (C_{1s} = 284.6 eV) was utilized as a reference for all binding energies calibration. The fitting of XPS results was performed using Multipack software. Inductively coupled plasma optical emission spectrometer (ICP) using iCAP6300 (Thermo-Fisher) was employed to determine Pt loadings (see Table. S1). Infrared spectroscopy (IR) measurements of the surface of different electrodes after soaking in 0.1M phosphate buffer containing 0.5mM NAD^+ for one hour, and then washed and dried were conducted with a Fourier transform IR spectrometer (INVENIO R, Bruker) equipping a liquid N_2-cooled HgCdTe detector and a diffuse reflectance infrared Fourier transform spectroscopy (DRIFTS) cell (Heat Chamber Type −1000°C, S.T. Japan, Inc.). This DRIFTS cell enables reflection absorption IR spectroscopy (RAIRS) by applying the electrode sample to its mirror-type sample holder having a ZnSe IR window. The ATR-FTIR measurement of crystalline NAD^+ was conducted with NICOLET, IS50 FT-IR with diamond crystal. Air was used as background, and the spectra were normalized of H_2O vapor (≈3900 cm^{-1}).

Table S1. Sample ID, and ICP results of Pt of the employed electrodes.

Sample ID	Pt conc (ppm)
TOT	0.0
**Pt-TOT.1**	3.0
**Pt-TOT.2**	4.0
**Pt-TOT.3**	5.5
**Pt-TOT.4**	7.0

Table S2. Elemental composition of different electrodes determined by SEM-EDS.

Sample ID	a=b (Å)	c (Å)	Cell volume (Å^3)
**TOT**	5.2	5.6	129.5
**Pt-TOT.1**	4.8	5.4	125.4
**Pt-TOT.2**	4.7	4.8	114.4
**Pt-TOT.3**	4.6	4.7	106.8
**Pt-TOT.4**	4.4	4.6	101.0

Table S3. Lattice parameters of different electrodes.

Sample ID	Elemental composition							
	Pt		Ti		O		C	
	Wt.%	At.%	Wt.%	At.%	Wt.%	At.%	Wt.%	At.%
**TOT**	0.0	0.0	66.6	39.5	31.4	55.8	2.0	4.6
**Pt-TOT.1**	14.8	2.8	64.9	49.6	19.0	43.5	1.3	4.1
**Pt-TOT.2**	17.1	3.1	56.2	40.2	24.4	52.2	1.6	4.5
**Pt-TOT.3**	30.7	5.7	42.7	32.2	23.3	52.5	3.2	9.7
**Pt-TOT.4**	38.9	7.9	37.8	31.1	19.3	47.5	4.1	13.6

Electrochemical studies. Electrochemical experiments were performed in a three-electrode system connected to a VersaSTAT3 potentiostat (Princeton Applied Research). A Ti mesh, TOT, **Pt-TOT**, Pt-Ti, Pt coil, and Pt sheet were used as a working electrode. A pure Pt coil with 230 mm length and 0.5 mm diameter (BAS Inc.) was employed as a counter electrode. A reference electrode (RE-1B) was composed of an Ag/AgCl electrode including 3 M NaCl (BAS Inc.). Cyclic voltammetry (CV) measurements were conducted using a three-electrode system constructed in a glass electrochemical cell. Electrochemical regeneration of 1,4- NADH and conversion of pyruvic acid to lactic acid were conducted in a three-electrode system employing a two-compartment cell (see Fig. S6) for 1 h using chronoamperometry, CA. An aqueous electrolyte solution of 0.1M phosphate buffer (pH 6) containing 0.5M NAD^+ was utilized for both CV measurements and all electrochemical 1,4-NADH regeneration experiments. For enzymatic formation of lactic acid 0.25 mM NAD^+ was used. Buffer solution without any NAD^+ was used for blank measurements. After introduction of the electrolyte solution into the cell, the system was tightly sealed. To provide an oxygen free medium, Ar gas was purged through the solution prior and during the measurements. The current value was recorded against the applied potential at a scan rate of 10 mV/s with 3 scan cycles for CV measurements. The applied potentials to the working electrodes were measured against the reference electrode (Ag/Ag/Cl) and converted to the reversible hydrogen electrode (RHE) for clarification using the following equation:

$$E \text{ (vs RHE)} = E \text{ (vs Ag/Ag/Cl)} + 0.196 \text{ V} + 0.059 \text{ V x pH} \quad \text{(S1)}$$

The Faradaic efficiency (FE) for the formation of NADH was calculated by Equation (S2):

$$FE = n_{NADH} n F / Q \quad \text{(S2)},$$

where n_{NADH} is the moles of the NADH produced measured by UV spectrophotometry, n represents the number of electrons required for the formation of one molecule of NADH (n = 2), F is Faraday's constant (96485 C mol^{-1} of electrons) and Q is the total charge in Coulomb passed across the electrode during the electrolysis.

For the calculation of Faradaic efficiency by the UV method, first calibration curves for both NAD^+ and 1,4-NADH were made using different concentrations as follows:

**NAD^+ concentration (mM)**	**1,4-NADH concentration (mM)**
0.05	0.01
0.1	0.05
0.15	0.1
0.2	0.2

Then the slopes were calculated and the concentration of our electrochemical regeneration products were calculated by equation S3 and shown in Fig. S7:

$$Y = \text{slope} * \text{concentration (mM)} + \text{int} \quad \text{(S3)}$$

In order to detect the concentration of the produced 1,4-NADH from our electrochemical reduction of NAD^+, the absorption peaks were detected at 340 nm by UV spectrophotometry. However, as shown in Scheme 1 (main text), 1,4-NADH exhibits a UV absorption peak around 340 nm and UV absorptions for possible byproducts such as $(NAD)_2$ dimer, 1,6-NADH are observable at similar positions such as 340 and 345, respectively. Thus, to precisely calculate the Faradaic efficiency for 1,4-NADH only, we detected the UV absorption peaks before and after the enzymatic assay and the difference between them was considered to be the concentration of the enzymatically active form 1,4-NADH.

The electrochemical double-layer capacitance (C_{dl}) was measured by the CV technique within the potential range from -0.2 to 0.4 V vs Ag/AgC at the changed scan rates from 10 to 100 mVs^{-1}. The C_{dl} values were determined by plotting halves of the differences between negative and positive current densities, the charging current (using equation S4) versus scan rates, and calculating the slope as shown in equation S5.

$$I_c[A] = (I_{anodic} - I_{cathodic})_{OCP} \quad \text{(S4)}$$

$$\text{Slope} = C_{dl}[F] = \Delta I_c[A]/\Delta \upsilon[Vs^{-1}] \quad \text{(S5)}$$

Finally, ECSA can be calculated utilizing specific charge density, c, of 40 μFcm^{-2} in equation 6 [2].

$$ECSA[cm^{-2}] = C_{dl}[F]/ c[\mu Fcm^{-2}] \quad \text{(S6)}$$

HER measurements were also conducted with the same conditions of the electrochemical regeneration of 1,4-NADH using Micro GC Fusion (INFICON). Electrochemical regeneration of 1,4- NADH was repeated using 0.1M D_2O phosphate buffer containing 4mM NAD^+ (pH6) for molecular analysis by ^1H-NMR spectroscopy in order to confirm the possible formed by-products using different electrodes. The results were compared with commercial NAD^+, 1,4-NADH, and the reported work. The analysis was conducted on NMR (JEOLJNM-ECS 400) at room temperature.

Enzymatic assay. After the electrochemical regeneration reaction, the reduced cofactor concentration was detected by UV spectrophotometry at 340 nm. However, as shown in scheme. 1(main text) both active 1,4- NADH and inactive 1,6- NADH show absorbance peaks at similar wavenumber, 340 and 345 nm, respectively. Therefore, the purity of the regenerated enzymatically active 1,4-NADH, should be obtained via the activity assay. Consequently, the activity assay described by [3] with some changes has been used to determine the enzymatic activity of the regenerated NADH (see scheme S1) according to converting acetaldehyde to ethanol using alcohol dehydrogenase following reaction (7) below [3]. As a result, a reduced absorbance at 340 nm can be clearly detected due to the1,4-NADH oxidation that is proportional to the real quantity of the enzymatically active 1,4-NADH.

$$\text{1,4-NADH} + \text{Acetaldehyde} + \text{ADH} \rightarrow NAD^+ + \text{Ethanol} \quad \text{(S7)}$$

To measure the enzymatic activity, 30µl of substrate (acetaldehyde) was introduced into 1 mL of regenerated 1,4-NADH in a cuvette. Then, the absorbance of the solution at 340 nm was recorded by a UV-vis spectrophotometer, until reaching a steady state value. A volume of 200 µl (12.1U) of the enzyme alcohol dehydrogenase was then injected into the mixture while the absorbance was detected until reaching a final constant value, indicating that the entire amount of active 1,4-NADH produced during the electrolysis was occupied in the enzymatic reaction. Hence, the regeneration (purity/ yield) of enzymatically active 1,4-NADH formed in electrolysis process was calculated by equation S8 [4]:

$$\text{Regeneration (Yield) of 1,4-NADH (\%)} = \frac{100- ((A/A_0) \times 100)}{\text{Initial } NAD^+ \text{ purity (\%)}} \times 100 \quad \text{(S8)}$$

where A stands for the final absorbance and A_0 is the initial absorbance. For confirmation of reproducibility and reliability of the obtained results, all the NADH regeneration experiments and enzyme assay have been replicated for at least three times. The assay was also first calibrated utilizing commercial NADH.

Purification of Lactate Dehydrogenase (LDH) from *Lactobacillus acidophilus*. *Lactobacillus acidophilus* (Type strain DSM 20079) was cultured in TSB (tryptic soy broth) medium for 3 days at 30 °C. The cultured cells were harvested by centrifugation at 9000 ×*g* for 20 min. The harvested cells (20 g) were suspended in a 20 mM potassium phosphate (KP) buffer (pH 7.0) containing 1 mM dithiothreitol (DTT) and disrupted twice at 60 W for 2 min in an ice bath using an Ultrasonic Disruptor UD-200 (TOMY SEIKO Inc., Japan). The resulting extract was ultra-centrifuged at 140,000 ×*g* for 1 h using Optima L-90K Ultracentrifuge (Beckman Coulter Inc., USA). The supernatant was collected as a soluble cell extract.

The cell extract was then directly loaded onto a Q Sepharose High Performance (2.6 × 15 cm, GE Healthcare UK Ltd.) pre-equilibrated with 20 mM KP buffer (pH 7.0). Column chromatography was performed at 4°C using an AKTA-FPLC system (GE Healthcare UK Ltd., Buckinghamshire, UK). The LDH was eluted around 0.2 M concentration by a linear gradient of NaCl between 0 and 0.3 M at a flow rate of 8 mL/min. The resulting solution was loaded onto was loaded onto a hydroxyapatite (1.6 cm × 10 cm; Bio-Rad Laboratories Inc.), pre-equilibrated with 10 mM KP buffer (pH 7.0) containing 0.5 mM DTT (buffer A). The column was washed with 100 mL of buffer A at a flow rate of 4 mL/min. A gradient method was used which consisted of 200 mL of buffer containing a gradient of 10 to 400 mM KP. The fractionations of LDH eluted around 120 mM KP were adjusted to give a final concentration of 1.0 M ammonium sulfate. The enzyme solution was then applied onto a Phenyl Sepharose High Performance (1 cm × 5 cm; GE Healthcare UK Ltd.) pre-equilibrated with buffer A containing 1.0 M ammonium sulfate. After loading, the column was washed with 20 mL of the same buffer. LDH was eluted with a linear gradient of decreasing ammonium sulfate concentration 1.0 to 0 M ammonium sulfate a flow rate of 2 mL/min. The active fractions of LDH were eluted from 500 mM ammonium sulfate. Protein purity of the active pool was established by SDS-PAGE analysis using gels containing 12.5% [wt/vol] acrylamide with standard molecular weight markers (GE Healthcare UK Ltd.) after staining in a dye solution of Coomassie Brilliant Blue R-250 (Fig. S14.).

To assess a physiological role of LDH, the enzyme activity was assayed in 10 mM pyruvate and 0.2 mM NADH in 50 mM KP buffer (pH 7.0). The specific activity was expressed as units/mg of protein, where 1 unit is equivalent to the oxidation of NADH per minute using the molar absorption coefficient at 340 nm (ε_{340}) of 6,220 $M^{-1} \cdot cm^{-1}$. Thermostability and optimal reaction pH of the purified LDH was then determined by enzymatic assay at different pH values and temperatures (Fig. S15) after performing lowery protein assay [5].

Activation of LDH using our electrochemical regenerated NADH for conversion of pyruvic acid into lactic acid. After purification of LDH, it was added to our electrochemical system within the cell containing the starting NAD^+ solution and pyruvic acid (20mM). The reaction was conducted at potential -0.8v vs Ag/AgCl and pH 6. The produced lactic acid was detected by High Performance Liquid Chromatography, HPLC (LC-20AD, Shimadzu) after making a calibration curve for both pyruvic acid and lactic acid (Fig. S16). In addition, during the electrochemical reaction the product was tested every 15 minutes using UV to prove the consumption of produced NADH during the formation of lactic acid. For comparison, the same procedure was done for only the electrochemical regeneration of NADH. Moreover, in situ UV (Fig. S17) was also conducted to show the change of NADH produced over time with and without LDH and pyruvic acid (Fig. S18).

Scheme S1. (a) Suggested good electrode for electrochemical reduction of NAD^+, and (b) representative enzymatic assay process used in the current work.

a

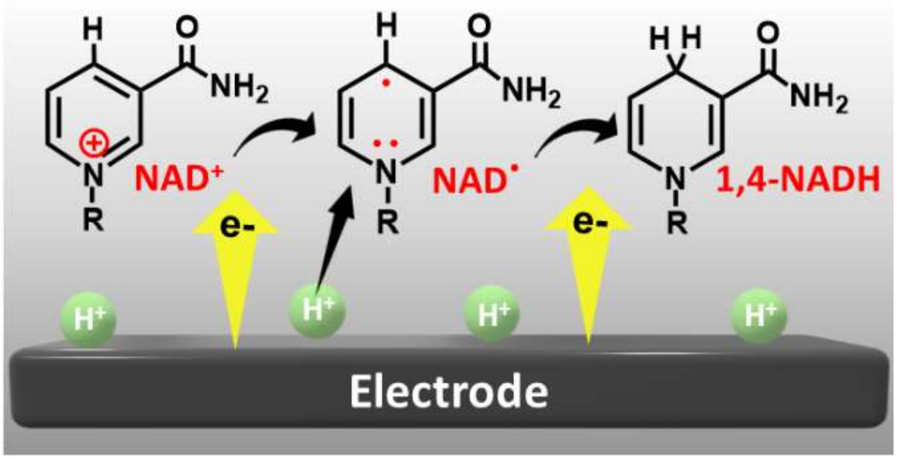

b

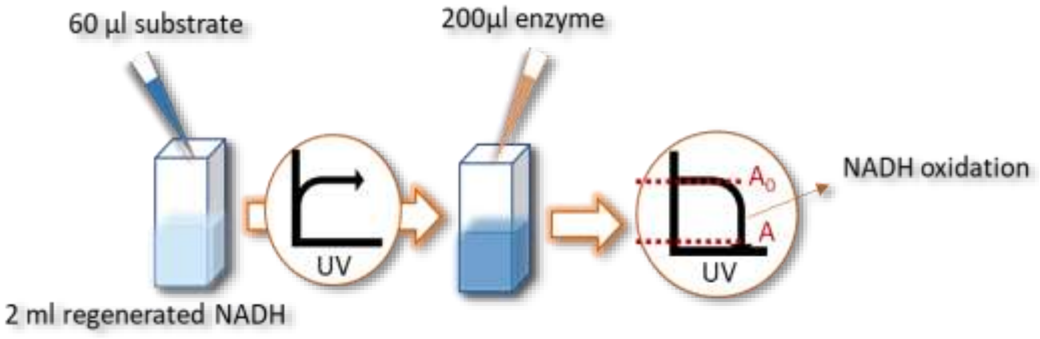

Substrate: acetaldehyde
Enzyme: alcohol dehydrogenase, ADH (121U/ mg powder)
Coenzyme: regenerated NADH
UV wavelength: 340 nm (the absorbance of the solution)

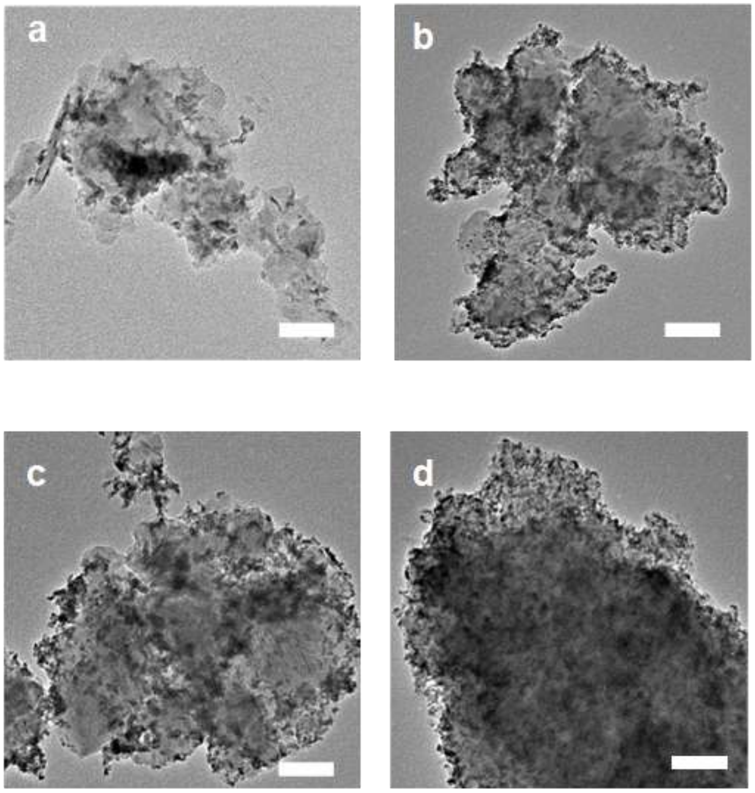

Fig. S1. TEM images and of Pt-TOTs with different Pt loading. (a) Pt-TOT.1, (b) Pt-TOT.2, (c) Pt-TOT.3, (d) Pt-TOT.4. The length of scale bars accords to 50 nm.

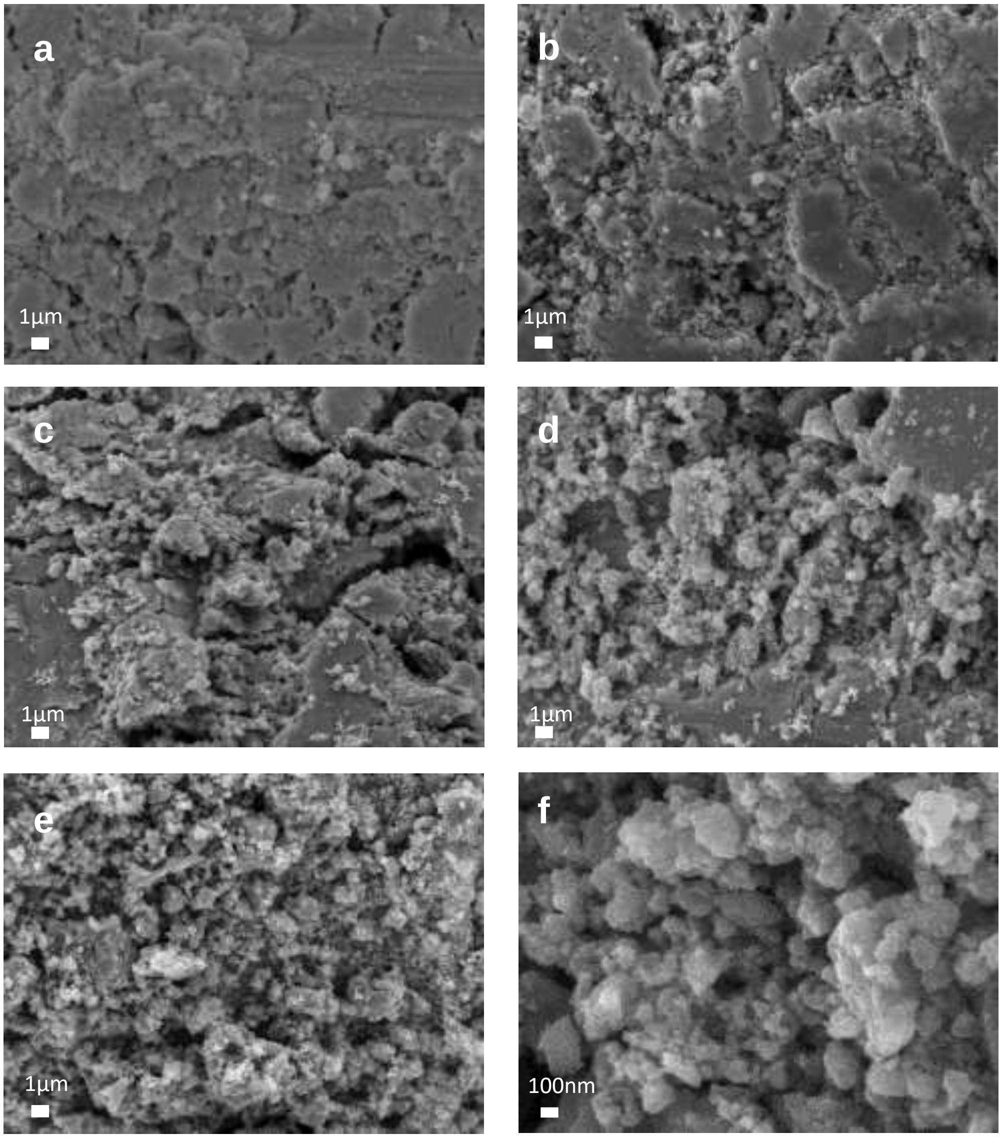

Fig. S2. SEM images of (a) TOT, (b) Pt-TOT.1, (c) Pt-TOT.2, (d) Pt-TOT.3, (e) Pt-TOT.4 and (f) high magnification images of Pt-TOT.2.

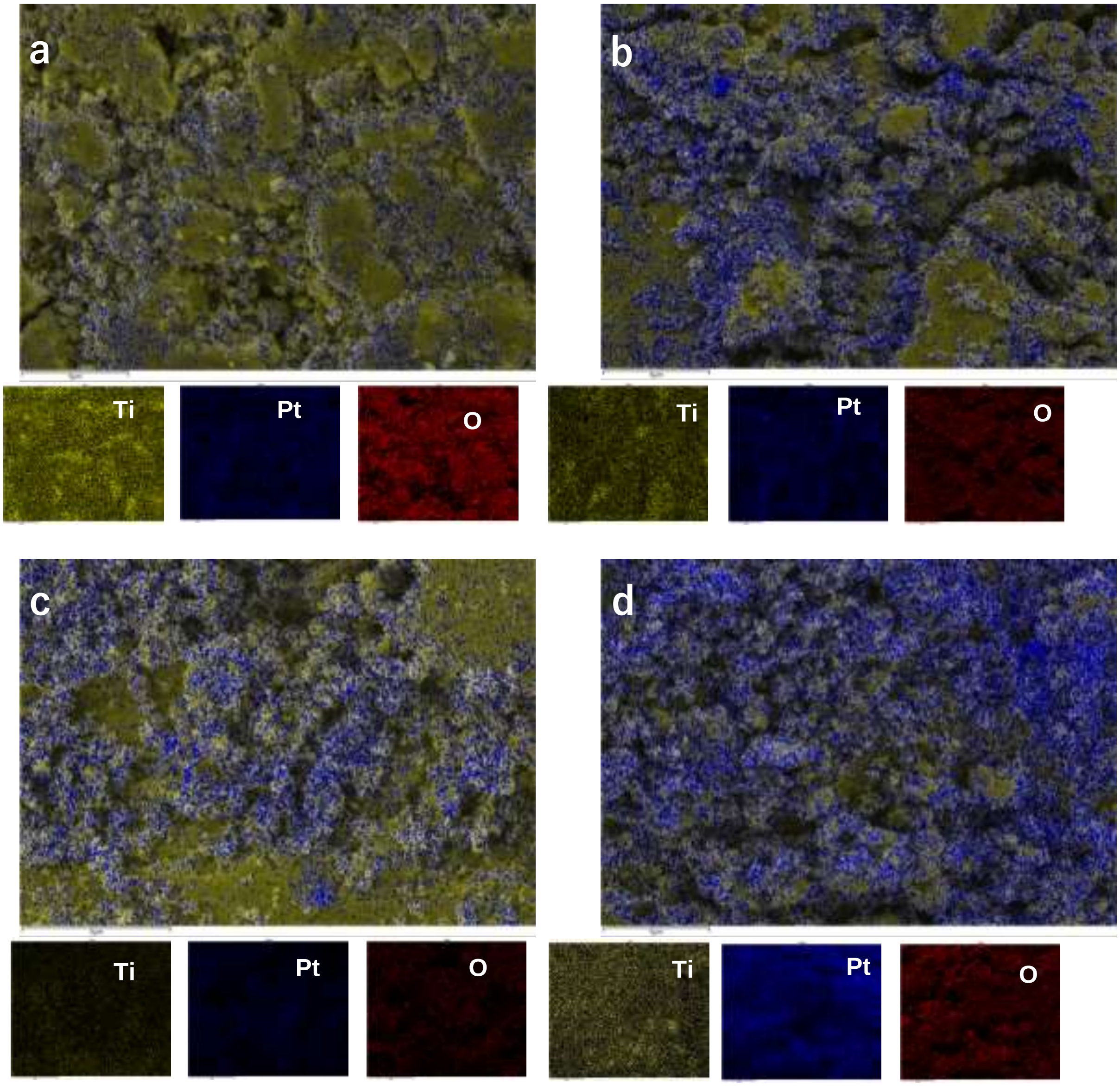

Fig. S3. SEM-EDS elemental mapping of (a) Pt-TOT.1, (b) Pt-TOT.2, (c) Pt-TOT.3 and (d)Pt-TOT.4.

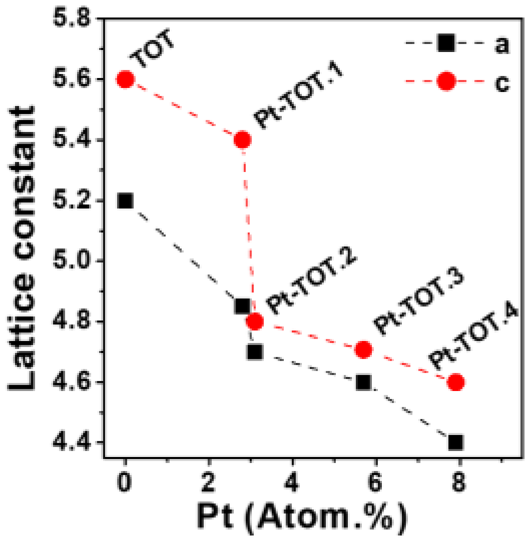

Fig. S4. Lattice constants (*a* and *c*) versus loading amounts of Pt of different electrode.

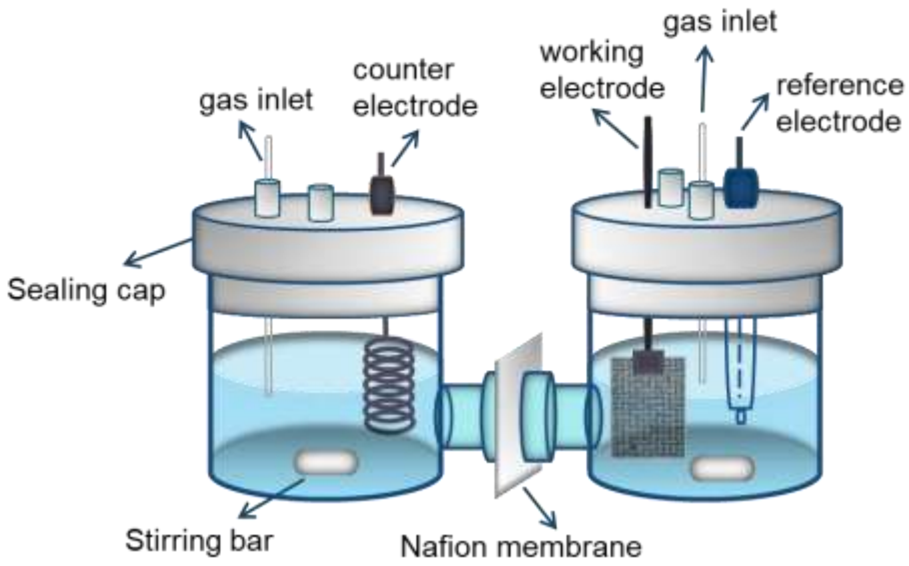

Fig. S5. Illustration of the two-compartment electrochemical cell (H-type cell) used for CA in this work.

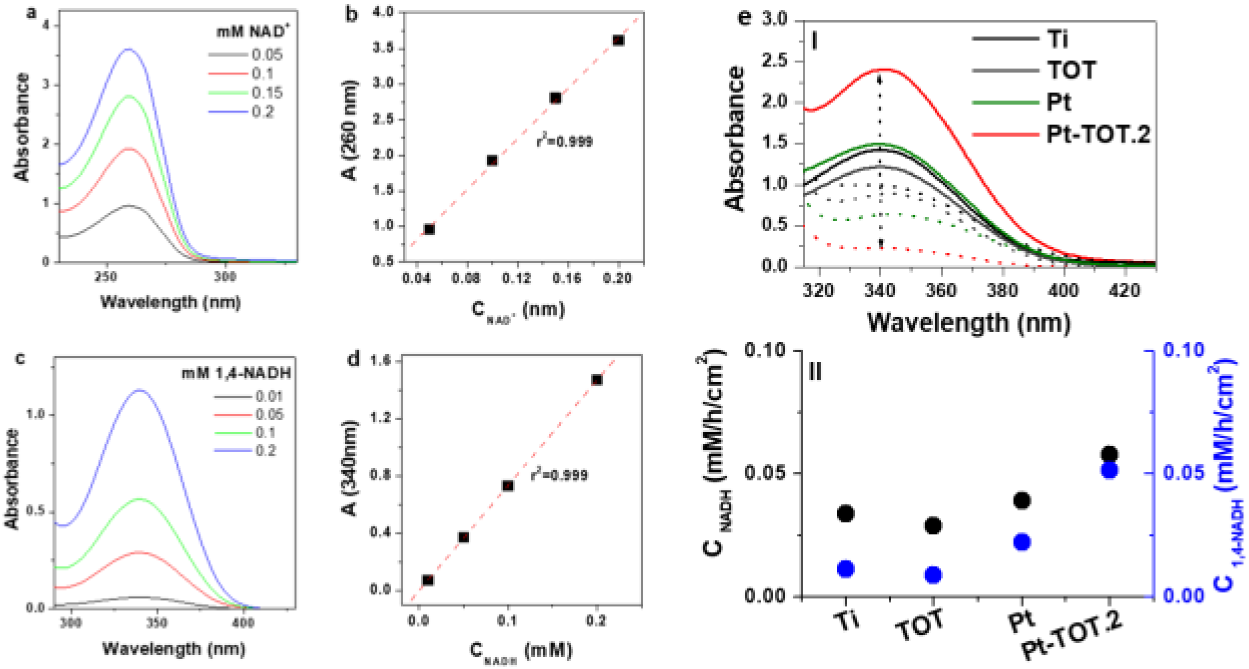

Fig. S6. UV absorbance and corresponding calibration curves with different concentrations for (a and b) NAD^+ and (c and d) for 1,4-NADH. (e.I) UV absorbance at 340 nm for electrochemical regenerated products on different electrodes at -1.2 V vs Ag/AgCl (solid and dashed lines are before and after enzymatic assay, respectively), the vertical dotted line represents the difference between before and after enzymatic assay, and (e.II) the corresponding production rates for NADHs (C_{NADH} and $C_{1,4\text{-}NADH}$) calculated from the number (mole) of totally produced NADH and 1,4-NADH per hour per electrodes' surface area.

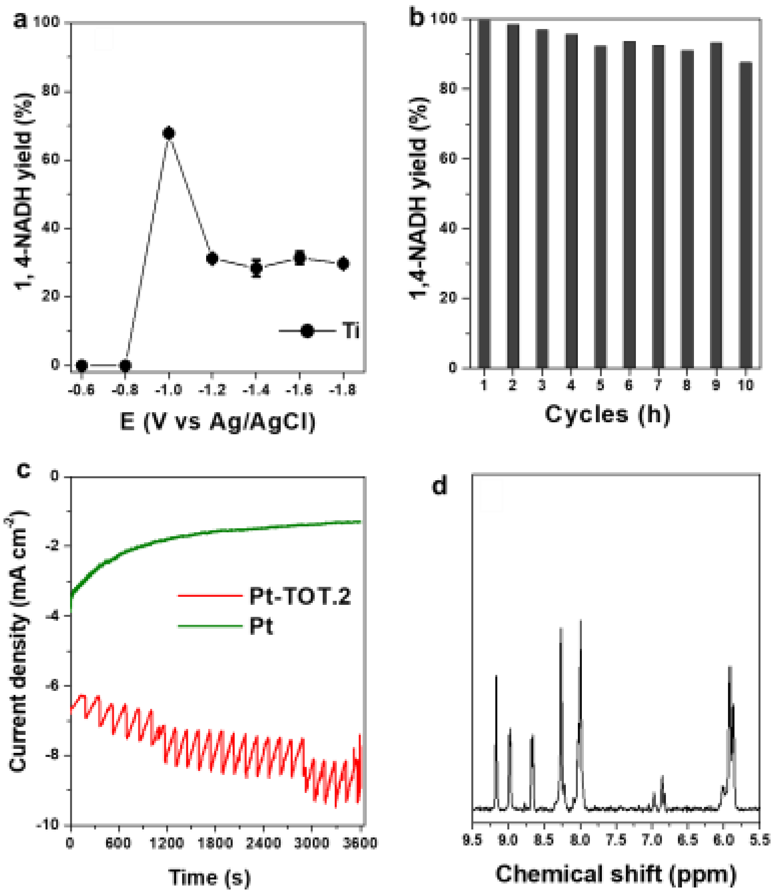

Fig. S7. (a) Yield for 1,4-NADH at various potentials on Ti, (b) the reusability of Pt-TOT.2 at -0.8V vs Ag/AgCl for number of cycles (each cycle is 1h), (c) chronoamperometry of Pt, and Pt-TOT.2 at -0.8V vs Ag/AgCl, and (d) ^{1}H NMR spectrum of products generated on Pt from 4 mM NAD^+ in 0.1M D_2O phosphate buffer with pH 6 at -1.2 V vs Ag/AgCl for 2h.

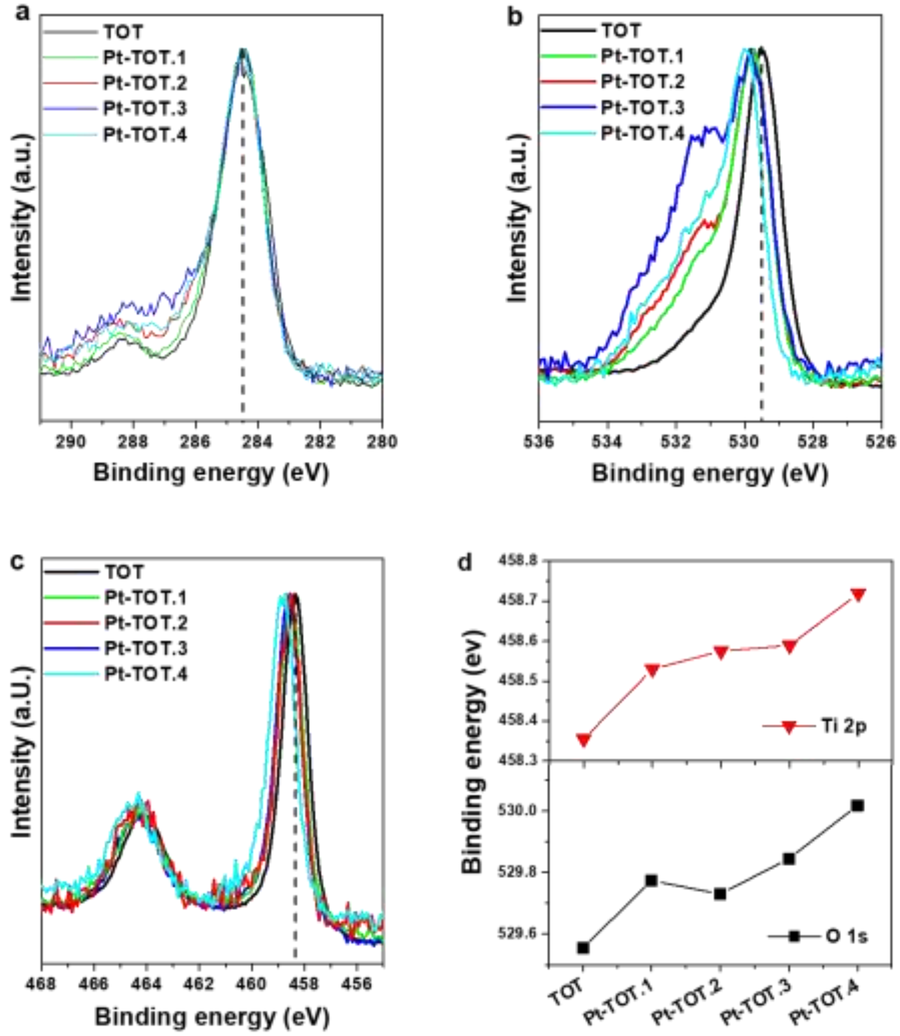

Fig. S8. XPS spectra of (a) C 1s, (b) O 1s, (c) Ti 2p, and (d) Shifts in binding energy for O 1s and Ti 2p of different electrodes.

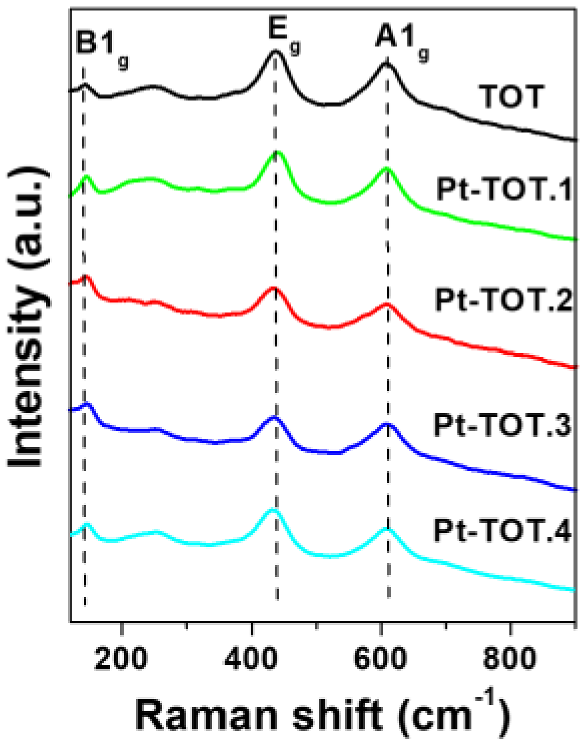

Fig. S9. Raman spectra of different electrodes.

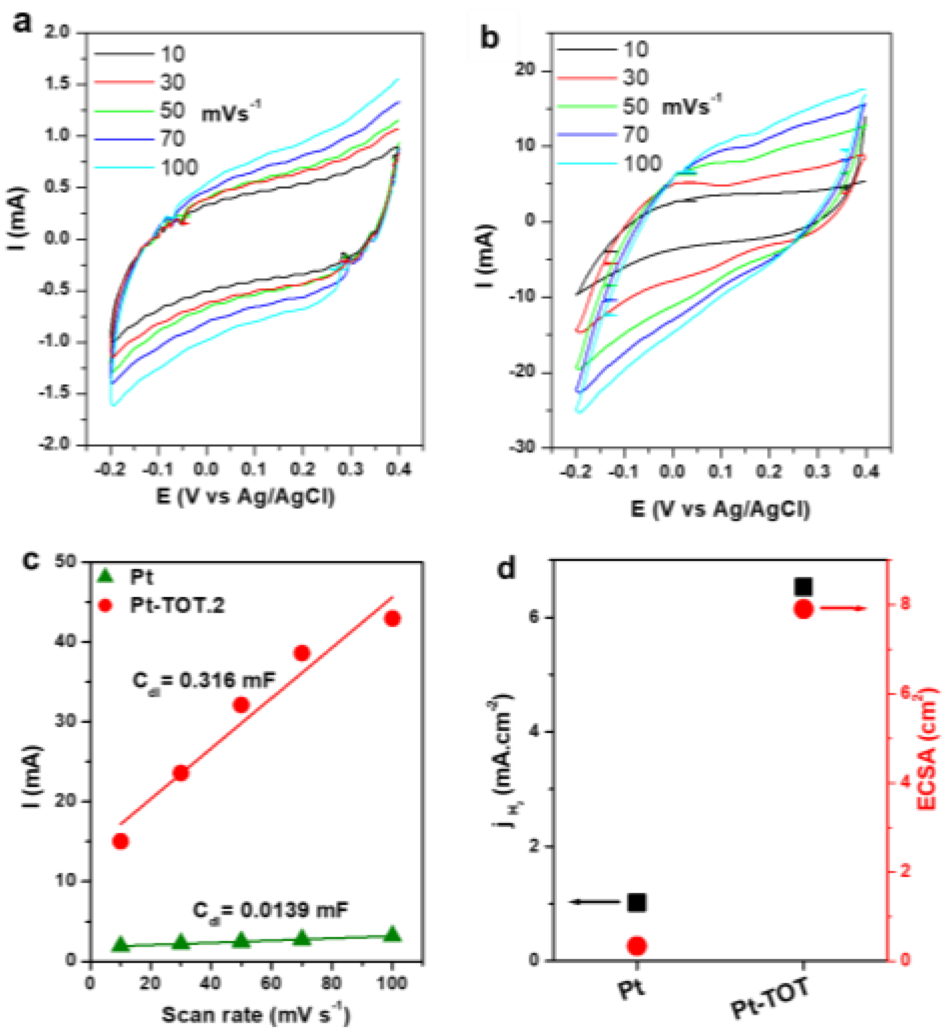

Fig. S10. Cyclic voltammograms on (a) Pt, and (b) Pt-TOT.2 at different scan rates from 10 to 100 mVs^{-1} within the potential window from -0.2 to 0.4 V vs Ag/AgCl, (c) Double layer capacitance, and (d) partial current density for HER and calculated electrochemical surface area on Pt, and Pt-TOT.2.

Table S4. Performance of the best up to date reported electrodes for direct electrochemical regeneration of 1,4-NADH.

Electrocatalyst	Reduction potential (V vs Ag/AgCl)	1,4-NADH yield (%)	Current/current density of LV/ CV	Reference
**Ti**	–1	96	0 mA	6
**$Ir_{0.8}Ru_{0.2}$-oxid/Ti**	–1.3	88	–10 mA	7
**Ni/MWCNT[a]**	–1.2	98	-	8
**Cu foam**	–1.1	80	–0.3 $mAcm^{-2}$	3
**CNF[b]**	–1.9	99.3	-	9
**Pt-GC[c]**	–1.2	100	-	10 (a)
**GC**	–3	98	–1 mA	10 (b)
**Pt-TOT[d]**	–0.6	86	–0.4 $mAcm^{-2}$	This work
**Pt-TOT[d]**	–0.8	99.5	–2.8 $mAcm^{-2}$	This work

a: Multiwalled Carbon Nanotubes

b: Carbon Nanofibers

c: Glassy Carbon

d: Pt^{n+} enhanced TiO_2/ Ti

Table. S5. XPS elemental analysis of different electrodes.

Sample name	Atomic concentration, %			
	C 1s	O 1s	Ti 2p	Pt 4f
**TOT**	30.83	50.35	18.81	0.00
**Pt-TOT.1**	45.34	33.16	8.98	12.44
**Pt-TOT.2**	37.01	32.83	9.46	20.70
**Pt-TOT.3**	43.31	23.31	6.01	27.37
**Pt-TOT.4**	39.67	24.14	4.66	31.53

It can be clearly seen that with increasing Pt content in Pt-TOT.x, the concentration of O decreases indicating formation of more defects of oxygen vacancy, Vo.

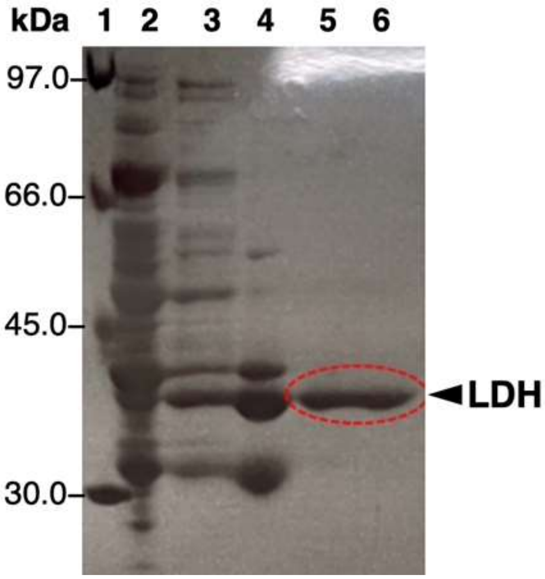

Fig. S11. SDS-PAGE (12.5%) of the purified LDH: Lane 1, low-molecular weight standard maker proteins (Mrs, 14,400 to 97,000); lane 2, cell-free extract; lane 3, the pooled fraction with LDH activity after Q Sepharose High Performance; lane 4, the pooled fraction with LDH activity after hydroxyapatite; lane 5-6, the pooled fraction with LDH activity after Phenyl Sepharose High Performance.

The molecular weight of the purified LDH was estimated as 150 ± 10 kDa using a calibrated gel filtration column of HiLoad® 16/600 Superdex® 200 pg (GE Healthcare UK Ltd.). The result suggests that the LDH is a homotetramer of molecular weight of 37 kDa.

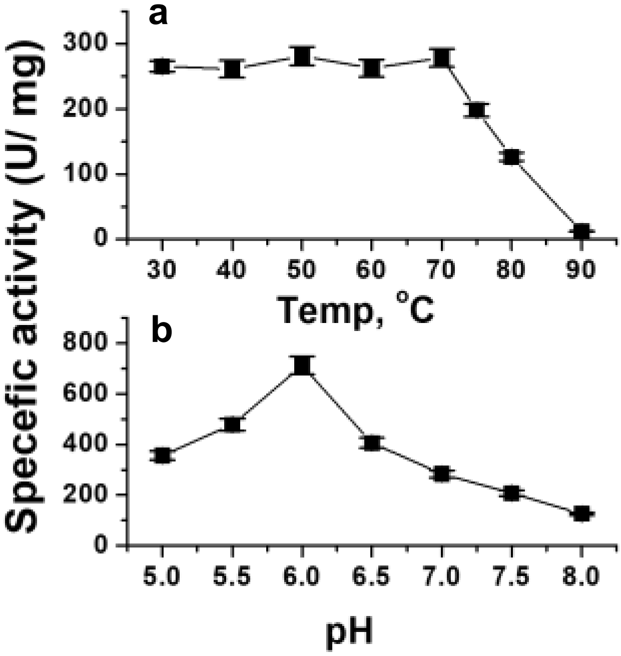

Fig. S12. Effects of temperature and pH on the LDH activity: (a) Thermostability determined at pH 7.0 over the temperature range from 30 to 90°C in 50 mM KP buffer, and (b) The optimal reaction pH of the purified LDH determined at 30°C in the pH range from 5.0 to 8.0 in 50 mM KP buffer.

The maximal activity of pH was observed at pH 6.0, but surprisingly, the activity was an extremely stable at thermal temperature up to 70°C and was gradually inactivated above the temperature.

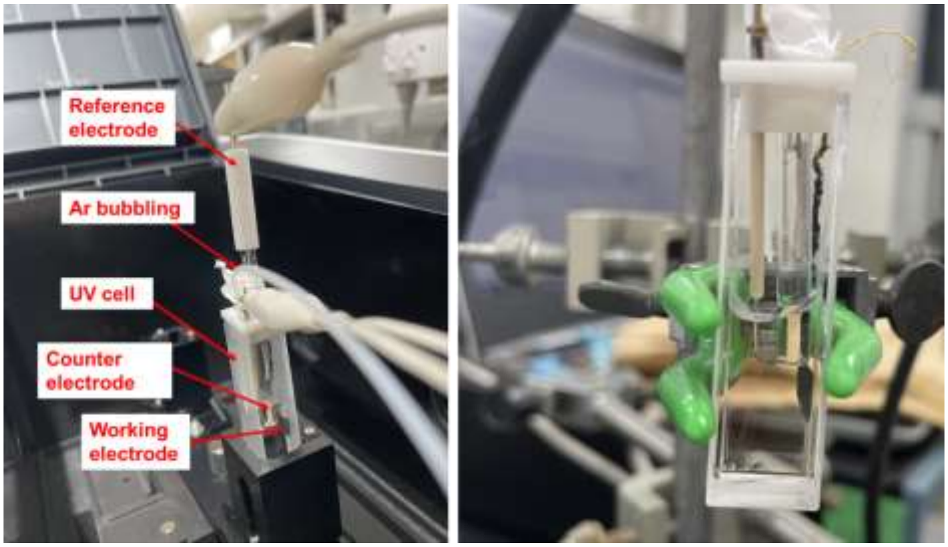

Fig. S13. In situ UV cell construction for electrochemical regeneration of NADH and conversion of pyruvic acid to lactic acid. Ag/AgCl, Pt rode, and a piece of Pt-TOT.2 electrode were used as reference electrode, counter electrode, and working electrode, respectively. Ar gas was purged for 25 Minutes before and during the reaction. The cell was connected to a VersaSTAT3 potentiostat (Princeton Applied Research).

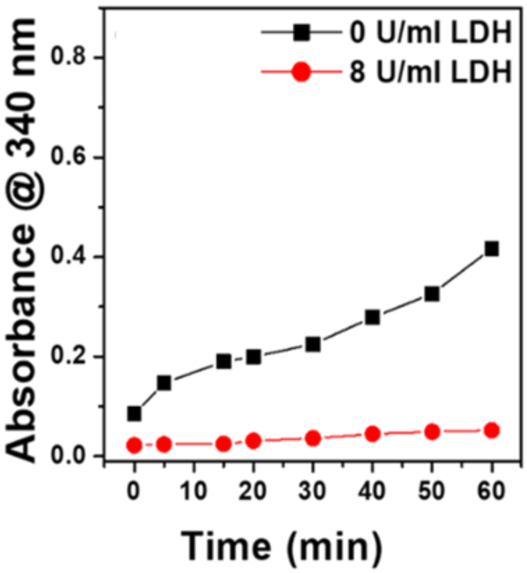

Fig. S14. In situ UV absorbance at 340 nm vs time for the electrochemical reduction of 0.25mM NAD^+, and 0.25mM NAD^+ with 20 mM pyruvic acid and 8 U/ml LDH.

Time course of adsorption intensity at 340 nm, which is originated from LDH, clearly shows the behavior in in situ UV measurements. It can be also noticed that the produced amount of NADH in the in situ UV cell (Fig. S17) was a little lower than that in ex situ measurement using a conversional two compartment cell (Fig. 6) due to the re-oxidation of NADH in an unseparated counter electrode.

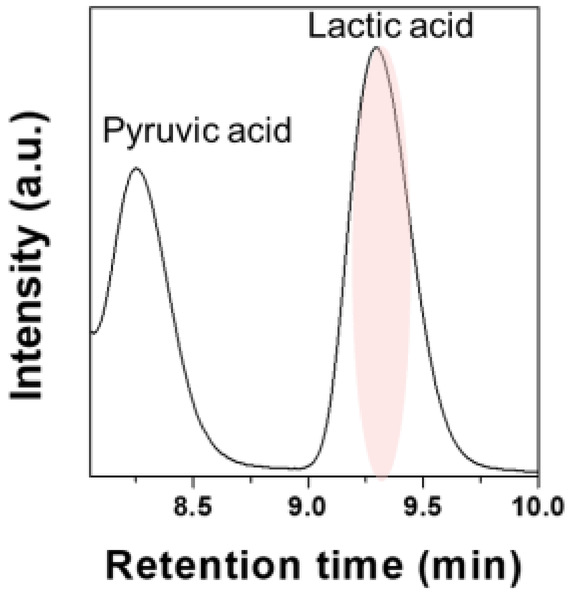

Fig. S15. The HPLC spectra of products of the electrochemical reduction of NAD^+ (0.25m) combined with conversion of pyruvic acid (20mM) to lactic acid using LDH (8 U/ml) at -0.8 V vs Ag/AgCl. The spectrum peaks observed at 8.25 and 9.3 min are originated from pyruvic acid and lactic acid, respectively.

Results and discussion

Mechanistic understanding

The Ti $2p_{3/2}$ and $2p_{3/2}$ XPS spectra of TOT and **Pt-TOT**s exhibited peaks around 458 and 464 eV, respectively, which were attributed to overlapping signals of Ti^{4+} and Ti^{3+} species (Figs. 4a, and S8c) [11]. Interestingly, Figs. 4a and S8 revealed that increasing Pt content in **Pt-TOT**s resulted in a slight positive shift of the peaks of both Ti 2p and O 1s signals compared to those on TOT [11]. This shift probably indicates the interaction of Pt species with Ti and O, forming Ti–O–Pt bonds and suggesting an increase in defects (Ti^{3+} and oxygen vacancy, V_o) as presented in Table S5.

The XPS peaks observed at 71.2 and 74.4 eV, 72.1 and 75.5 eV, 73.7 and 77.1 eV, and 75.6 and 78.9 in the Pt 4f spectra of **Pt-TOT.2** (Fig. 4b) were attributed to Pt^0 ($4f_{7/2}$ and $4f_{5/2}$), Pt^{2+} ($4f_{5/2}$ and $4f_{7/2}$), Pt^{3+} ($4f_{7/2}$ and $4f_{5/2}$), and Pt^{4+} ($4f_{7/2}$ and $4f_{5/2}$), respectively [12], confirming the presence of Pt^{n+} species with different valence states in **Pt-TOT.2**.

Consistent with the XRD results, Raman spectra of TOT and **Pt-TOT**s exhibited three prominent peaks around 144.1, 446.1, and 608.4 cm^{-1}, corresponding to B1g, Eg, and A1g modes of rutile, respectively (Figs. S9, and 4c) [12].

Hydrogen evolution behavior of different electrodes:

It is generally known that HER can be proceed through three steps as follow [13]:

(a) Electrochemical hydrogen adsorption (Volmer reaction),

$H^+ + M + e \leftrightarrow MH_{ads}$ (S9)

Or

$H_2O + M + e \leftrightarrow MH_{ads} + OH^-$. (S10)

(b) Electrochemical desorption (Heyrovsky reaction)

$MH_{ads} + H^+ + e \leftrightarrow M + H_2$ (S11)

Or

$MH_{ads} + H_2O + e \leftrightarrow M + H_2$ (S12)

(c) Chemical desorption (Tafel reaction)

$2MH_{ads} \leftrightarrow 2M + H_2$ (S13)

The protonation of NAD^+ is a key for production of more enzymatically active 1,4-NADH. According to HER results, **Pt-TOT**s were possibly covered with larger amounts of H_{ads} during the HER, which is favorable for increasing the kinetic of Step 2a in Scheme 1 (the main text).

Proposed mechanism on Pt-TOT:

The findings presented in this study shed light on the mechanism underlying NADH-reg on Pt-TOTs, and we propose the following mechanism based on our results:

$\text{Pt-TOT} + NAD^+ \leftrightarrow \text{Pt-TOT} + NAD^+_{ads}$ (S14)

$\text{Pt-TOT} + H_2O + e^- \leftrightarrow \text{Pt-TOT} + H^*_{ads} + OH^-$ (S15)

$\text{Pt-TOT} + NAD^+_{ads} + e^- \leftrightarrow \text{Pt-TOT} + NAD^*_{ads}$ (S16)

$\text{Pt-TOT} + NAD^*_{ads} + H^*_{ads} \leftrightarrow \text{Pt-TOT} + NADH_{ads}$ (S17)

In the proposed mechanism, we designate "ads" and "*" to symbolize the adsorption of NAD^+ on Pt-TOT and the existence of a radical state on the surface, respectively. Equations S14 and S15 describe the adsorption and reduction of NAD^+ on **Pt-TOT**, while the subsequent hydrogenation of the adsorbed NAD occurs rapidly through a reaction with $H*_{ads}$ formed around Pt sites, as illustrated in Equations S16 and S17 and Scheme 2b in the main text. Alternatively, we propose the following Equations S18 and S19 as an alternative pathway to replace Equations S16 and S17.

$$\text{Pt-TOT} + NAD^+_{ads} + H^*_{ads} \leftrightarrow \text{Pt-TOT} + NADH^{*+}_{ads} \quad (S18)$$

$$\text{Pt-TOT} + NADH^{*+}_{ads} + e^- \leftrightarrow \text{Pt-TOT} + NADH_{ads} \quad (S19)$$

References

[1] Z. LIan, W. Wang, G. Li, F. Tian, K.S. Schanz, H. Li, Pt-Enhanced Mesoporous Ti^{3+}/TiO_2 with Rapid Bulk to Surface Electron Transfer for Photocatalytic Hydrogen Evolution, ACS Appl. Mater. Interfaces 2017, 9, 16959-16966.

[2] E. Cossar, M. S.E. Houache, Z. hang, E.A. Baranova, Comparison of electrochemical active surface area methods for various nickl nan0structures, J. Elect. Chem. 2014, 870, 114246.

[3] R. Barin, S. Rashid-Nadimi, D. Biria, M.A. Asadollahi, Direct electrochemical regeneration of 1,4-NADH at the copper foam and bimetallic copper foam, Electrochem. Acta 2017, 247, 1095-1102.

[4] I. Ali, T. Khan, S. Omanovic, Direct electrochemical regeneration of the cofactor NADH on bare Ti, Ni, Co and Cd electrodes: The influence of electrode potential and electrode material, J. Molec. Catal. A: Chemical 2014, 387, 86–91.

[5] Lowery, OH, NJ Rosbrough, AL Farr, RJ Randall, J. Biol. Chem. 1951, 193, 265.

[6] I. Ali, T. Khan, S. Omanovic, Direct electrochemical regeneration of the cofactor NADH on bare Ti, Ni, Co and Cd electrodes: The influence of electrode potential and electrode material, J. Molec. Catal. A: Chem. 2014, 387, 86-91.

[7] N. Ullah, I. Ali, S. Omanovic, Direct electrocatalytic reduction of coenzyme NAD^+ to enzymatically- active 1,4- NADH employing an iridium/ruthenium-oxide electrode, Mater. Chem. Phys 2015, 149-150, 413-417.

[8] I. Ali, N. Ullah, M. A. McArthur, S. Coulombe, S. Omanovic, Direct Electrochemical Regeneration of Enzymatic Cofactor1,4-NADH on a Cathode Composed of Multi-Walled Carbon Nanotubes Decorated with Nickel Nanoparticles, Can. J. Chem.Eng.2018, 96, 68-73.

[9] I. Ali, M. McArthur, N. Hordy, S. Coulombe, S. Omanovic, Electrochemical regeneration of the cofactor NADH employing a carbon nanofibers cathode, Int. J.Electrochem.Sci.2012, 7, 7675-7683.

[10] (a) I. Ali, A. Gill, S. Omanovic, Direct electrochemical regeneration of the enzymatic cofactor 1,4-NADH employing nano-patterned glassy carbon/Pt and glassy carbon/Ni electrodes, Chem. Eng. J 2012, 188, 173-180. (b) I. Ali, B. Soomro, S. Omanovic, Electrochemical regeneration of NADH on a glassy carbon electrode surface: The influence of electrolysis potential, Electrochemistry Communications 2011, 13, 562–565.

[11] Z. Lian, W. Wang, G. Li, F. Tian, K.S. Schanze, H. Li, Pt-Enhanced Mesoporous Ti^{3+}/TiO_2 with Rapid Bulk to Surface Electron Transfer for Photocatalytic Hydrogen Evolution, ACS Appl. Mater. Interfaces 2017, 9, 16959-16966.

[12] (a) O. Frank, M. Zukalova, B. Laskova, J. Kürti, J. Koltai, L. Kavan, Raman spectra of titanium dioxide (anatase, rutile) with identified oxygen iso-topes (16, 17, 18), Phys. Chem. Phys. 2012, 14, 14567-14572. (b) P. S. Narayanan, Raman spectrum of rutile, Polarisation studies, Department of Physics, lndian lnstitute of Science, Bangalore 3, 1953.

[13] R. Subbaraman, D. Tripkovic, D. Strmcnik, Kee-Chul Chang, et al., Enhancing Hydrogen Evolution Activity in Water Splitting by Tailoring Li^+-$Ni(OH)_2$-Pt Interfaces, Science 2011, 334, 1256.
